# Unraveling Element-Selective Local Structures in Multielement Alloy Nanoparticles with EXAFS

**DOI:** 10.1021/acsnanoscienceau.5c00013

**Published:** 2025-04-30

**Authors:** Masashi Nakamura, Dongshuang Wu, Megumi Mukoyoshi, Kohei Kusada, Hiroyuki Hayashi, Takaaki Toriyama, Tomokazu Yamamoto, Yasukazu Murakami, Hirotaka Ashitani, Shogo Kawaguchi, Toshiaki Ina, Osami Sakata, Yoshiki Kubota, Isao Tanaka, Hiroshi Kitagawa

**Affiliations:** † Division of Chemistry, Graduate School of Science, 12918 Kyoto University, Kitashirakawa-Oiwakecho, Sakyo-ku, Kyoto 606-8502, Japan; ‡ The HAKUBI Center for Advanced Research, Kyoto University, Kitashirakawa-Oiwakecho, Sakyo-ku, Kyoto 606-8502, Japan; § Department of Materials Science and Engineering, Graduate School of Engineering, Kyoto University, Yoshida-Honmachi, Sakyo-ku, Kyoto 606-8501, Japan; ∥ The Ultramicroscopy Research Center, Kyushu University, 744 Motooka, Nishi-ku, Fukuoka 819-0395, Japan; ⊥ Department of Applied Quantum Physics and Nuclear Engineering, Graduate School of Engineering, Kyushu University, 744 Motooka, Nishi-ku, Fukuoka 819-0395, Japan; # JapanSynchrotron Radiation Research Institute (JASRI), SPring-8, 1-1-1 Kouto, Sayo-cho, Sayo-gun, Hyogo 679-5198, Japan; ∇ Department of Physics, Graduate School of Science, Osaka Metropolitan University, 1-1 Gakuen-cho, Naka-ku, Sakai, Osaka 599-8531, Japan

**Keywords:** multielement alloy, high-entropy alloy, platinum-group metal, *p*-block metal, EXAFS, element-selective structure, dynamic structure

## Abstract

We demonstrate physically consistent and interpretable extended X-ray absorption fine structure (EXAFS) curve-fitting analyses for estimating element-selective local structures in multielement alloy nanoparticles (MEA NPs). The difficulty in analyzing multielement systems originates from the too large number of independent structural parameters to fit, far exceeding the information content of the typical experimental data. Herein, this challenge is overcome by simultaneously fitting multiple data at different absorption edges and temperatures while imposing constraints based on a physically reasonable model. Another advantage of our approach is interpretability; the individual contributions of the constituent elements to the static and dynamic structures are explicitly estimated as atomic radii and Einstein temperatures. This method is used to analyze MEA NPs composed of platinum-group metals and *p*-block metals, which have contrasting properties, including atomic radii, melting points, and electronegativities. The results indicate that the local structures reflect the intrinsic nature of the elements and are also influenced by the interactions among them. The local structures around the *p*-block metals in the MEA NPs are shown to be distinctively modulated compared with those in the corresponding monometals, which is attributed to the electronic interaction with the platinum-group metals based on *ab initio* calculations. Our method is expected to facilitate the experimental characterization of these structurally complicated nanomaterials, which have been analyzed relying on calculations, yielding more precise pictures of real systems for investigating structure–property relationships.

## Graphical abstract

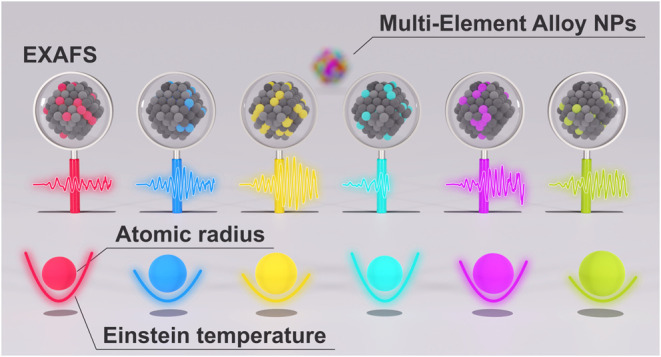

Recent progress in the automation of experiments and materials informatics has expanded the material search space, attracting greater attention to structurally complicated materials. One remarkable example is multielement alloy nanoparticles (MEA NPs), including high-entropy alloy NPs, which are characterized by a disordered atomic arrangement of multiple elements and nanoscale crystals. Since the late 2010s, MEA NPs have been extensively studied in the field of catalysis, demonstrating excellent activities and durabilities that surpass those of simpler alloy NPs.^1–3^ Although the origin of these superior properties remains controversial because of the structural complexity of MEA NPs, the local variety in their structures appears to play a crucial role.^3^ For example, lattice strain arising from the coexistence of elements with various atomic radii has been reported to lower the diffusion coefficients, kinetically preventing the structural deactivation of catalysts.^4,5^ Additionally, this local structural variety may create active sites with optimum adsorption energies.^6–9^ However, these structural insights have primarily emerged from computational studies, highlighting the need for experimental validation of the element-selective local structures in MEA NPs. The current crystal structure analyses of MEA NPs face several limitations.^10^ The commonly used X-ray diffraction (XRD) analysis only provides average structures and therefore is insufficient for examining the structural diversity. While advanced scanning transmission electron microscopy (STEM) techniques, such as 4D-STEM^11^ and atomic-resolution tomography,^12,13^ can reveal the local variety of structures because of their high spatial resolution, they struggle to differentiate all elements in the alloys. Given that these scattering-based techniques distinguish elements only based on their scattering factors, which are closely related to the atomic numbers, they are poorly suited for differentiating the constituent elements of MEA NPs, where elements with similar atomic numbers and thus scattering factors commonly coexist.

Extended X-ray absorption fine structure (EXAFS) offers a compelling alternative, providing information on the local structures around specific elements owing to the element-selectivity of core-level spectroscopy, which is ideal for untangling the complexity of MEA NPs. Furthermore, EXAFS curve-fitting enables numerical estimation of structural parameters, including bond lengths and their static and dynamic fluctuations. Notably, EXAFS can evaluate dynamic structures that remain inaccessible to STEM techniques, having been extensively exploited to study the atomic dynamics and interatomic potentials that can be deduced from dynamics.^14^ The applications of EXAFS range from ionic motions in superionic conductors^15^ and lattice behaviors around phase transitions^16,17^ to bond stiffness in nanomaterials.^18^ Despite these advantages, EXAFS curve-fitting of MEA NPs has remained underutilized, mainly because of the overwhelming number of structural parameters exceeding the information content of experimental data—a challenge unique to multielement systems.^19^ Previous attempts at the EXAFS curve-fitting of MEA NPs or bulk MEAs have managed this complexity by reducing the number of parameters by averaging structures,^4,20–26^ assuming perfectly disordered elemental distributions,^20,22^ or neglecting some scattering paths.^4,24^ For example, for an alloy formulated as ABCDE, the EXAFS oscillation at the absorption edge of element A arises from the backscattering of photoelectrons by A, B, C, D, and E. Although the number of parameters could be reduced by averaging the structures, namely by assuming that the structural parameters of all or some paths have common values, this approach sacrifices the information about structural diversity. In addition, physically inconsistent structures are often estimated; the structural parameters characterizing the path between elements A and B estimated by EXAFS at the absorption edges of these elements do not necessarily agree. Further improvements can be made to fully and precisely extract the structural information from experimental data.

Herein, we report the physically consistent and interpretable EXAFS analyses of MEA NPs. We overcome the traditional limitations by simultaneously fitting multiple data acquired at different absorption edges and temperatures while imposing constraints on fitting parameters based on a physically reasonable model. Moreover, the dynamic structures are evaluated by fitting the temperature dependence of the displacement parameters to the Einstein model taking advantage of the variable-temperature measurements. The derived Einstein temperatures represent lattice hardness and are crucial for understanding various lattice properties, including melting behavior and atomic diffusion.^14,27^ To demonstrate this method, we analyze MEA NPs composed of platinum-group metals (PGMs) and *p*-block metals (*p*Ms), which have markedly contrasting properties. The estimated parameters representing the element-selective static and dynamic local structures indicate that the local structures around a certain element reflect both its intrinsic elemental nature and the influences of interelemental interactions.

## Results and Discussion

### Synthesis and General Characterization of MEA NPs

First, the target MEA NPs composed of PGMs and *p*Ms were synthesized. These elements have contrasting properties,^28^ including atomic radii, melting points,^29^ mechanical properties,^30^ and electronegativities^31^ (Table S18). Therefore, their combination is expected to emphasize the variety of local structures. The syntheses were performed with a wet-chemical method employing oleylamine as the solvent and glucose as the reducing agent. Using this method, quinary alloy NPs comprising PGMs (Ru, Rh, Pd, Ir, and Pt) and a series of senary alloys with one additional *p*M (Ga, In, or Sn) were synthesized. Hereafter, these NPs are referred to as PGM MEA NPs and PGM–*p*M MEA NPs, respectively. While the precursor ratios among PGMs were always fixed equimolar, the fractions of *p*Ms were varied up to 25%. Eighteen samples with different compositions were synthesized via exactly the same procedure. Sample composition was determined using X-ray fluorescence (XRF) spectroscopy. All elements were incorporated despite a decrease in the fractions of Ga and In compared with those of the corresponding precursors (Table S1). This decrease can be attributed to the low reduction potentials of Ga and In ions. Transmission electron microscopy (TEM) analysis showed that all the samples comprised NPs with average sizes of 5–15 nm (Figures S1 and S2). Elemental distribution mapping performed using energy dispersive X-ray (EDX) spectroscopy coupled with STEM confirmed the presence of all elements in the NPs (Figures 1a and S3), strongly supporting alloy formation. In addition, chemical states were analyzed using X-ray photoelectron spectroscopy (XPS) and X-ray absorption near edge structure (XANES). In-depth XPS analysis using Ar-ion sputtering indicated that the *p*Ms were present in the metallic states inside the NPs, although oxides were present on the surface (Figure 1b and Table S2). More interestingly, the XANES spectra of the *p*Ms had spectral shapes distinct from those observed for the corresponding monometals, oxides, and their linear combinations (Figure 1c). This implies the unique electronic states of the *p*Ms resultant from their interactions with the PGMs. XANES data at absorption edges of PGMs also indicated that the PGMs were mainly in the metallic states (Figure S4). The chemical shifts and whiteline intensities of the XANES spectra were monotonically related to *p*M fractions, which can be attributed to electron transfer from *p*Ms to PGMs following their electronegativities (Figure S5). The consequence of such electronic interactions on alloy structures is an intriguing point to be discussed later.

**Figure 1:**
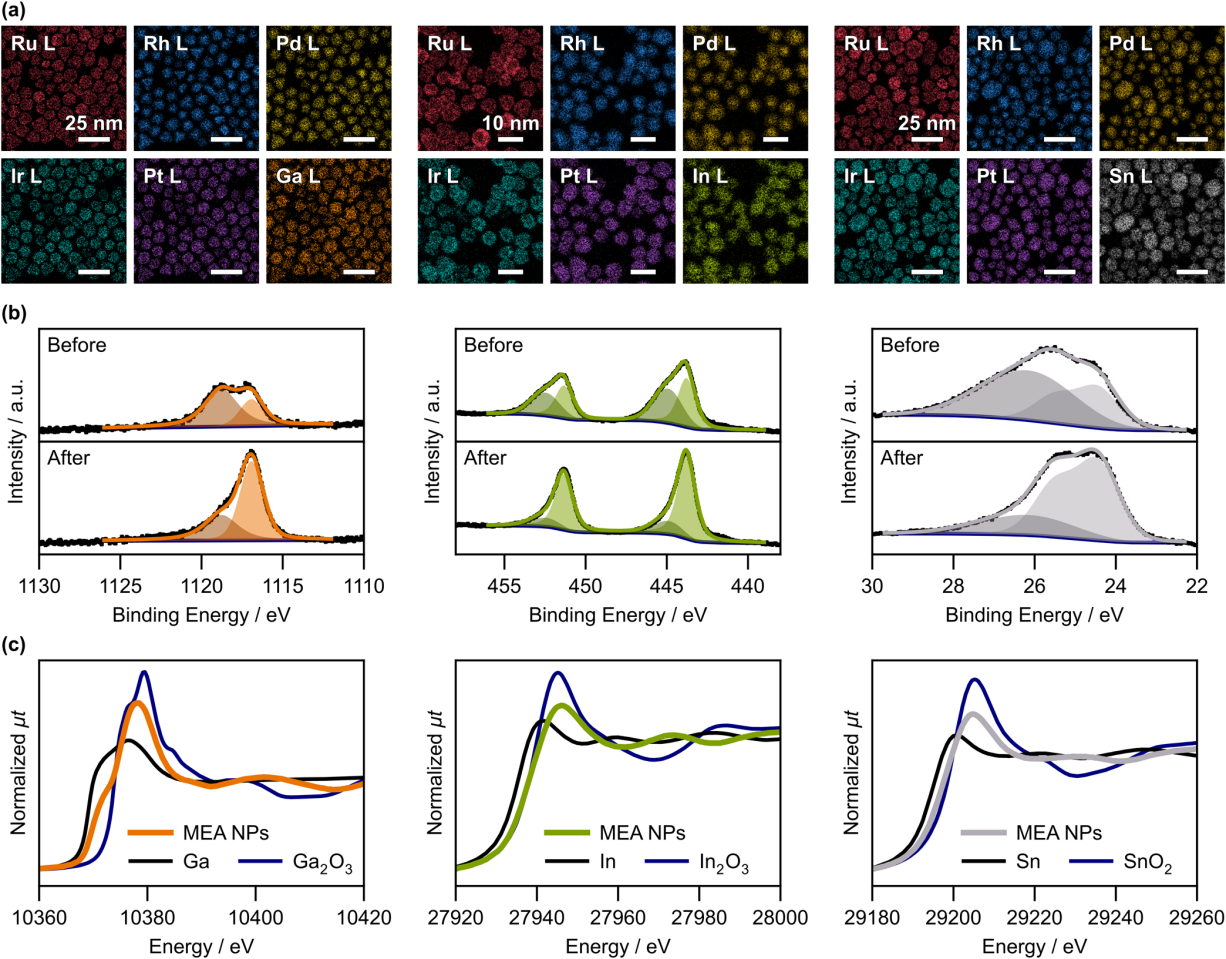
Characterization of *p*M–PGM MEA NPs (left: *p*M = Ga, center: *p*M = In, right: *p*M = Sn) synthesized using equimolar precursor ratios. (a) Elemental maps obtained by STEM–EDX. (b) XPS spectra of *p*Ms (left to right: Ga 2*p*_3/2_, In 3*d*, and Sn 4*d*) before (top) and after (bottom) Ar-ion sputtering. Black dots and colored lines are the experimental data and fitting curves, respectively. Spectra were deconvoluted into two peaks assigned to metal and oxide components (Table S2). (c) K-edge XANES spectra of *p*Ms in samples and references (monometals and common oxides).

### Average Structure Analyses by XRD

Prior to investigating the element-selective structures using EXAFS, we analyzed average structures using XRD. The relationships between compositions and structural parameters were examined to indirectly characterize the element-selective structures. Measurements were performed at the SPring-8 BL13XU beamline^32^ using an incident X-ray energy of 60 keV. Diffraction profiles were acquired at five temperatures between 105 and 300 K (Figures S6 and S7). The profiles of all samples could be assigned to monophasic face-centered cubic (fcc) structures. Rietveld refinement was then performed on all data assuming the monophasic fcc model using the GSAS-II software^33^ (Figure S8). The structural parameters including lattice parameters, microstrains, and displacement parameters were derived (Table S3). The temperature dependence of the displacement parameters was analyzed using the Einstein model to estimate the Einstein temperatures,^34,35^ which are indicators of lattice hardness (Figure S9, see Supporting Information Section S2.3 for details). Figure 2 shows the compositional dependences of the lattice parameters, microstrains, and Einstein temperatures, revealing that all parameters monotonically vary with the *p*M fraction. These trends reflect the properties of the *p*Ms (Tables S4 and S18). For example, the addition of In and Sn, whose radii are larger than those of PGMs,^29^ resulted in lattice expansion and increased strain. For PGM–Ga MEA NPs, the trend was difficult to predict, as the atomic radius of Ga strongly depend on the polymorph.^36–40^ Based on the above results, the radius of Ga was expected to be minimally smaller than the PGM average, giving rise to a minimal increase in microstrains. In addition, the decrease in Einstein temperatures with an increase in the *p*M fraction can be ascribed to the *p*Ms being softer than the PGMs, as represented by the corresponding melting points^29^ and mechanical properties.^30^

**Figure 2:**
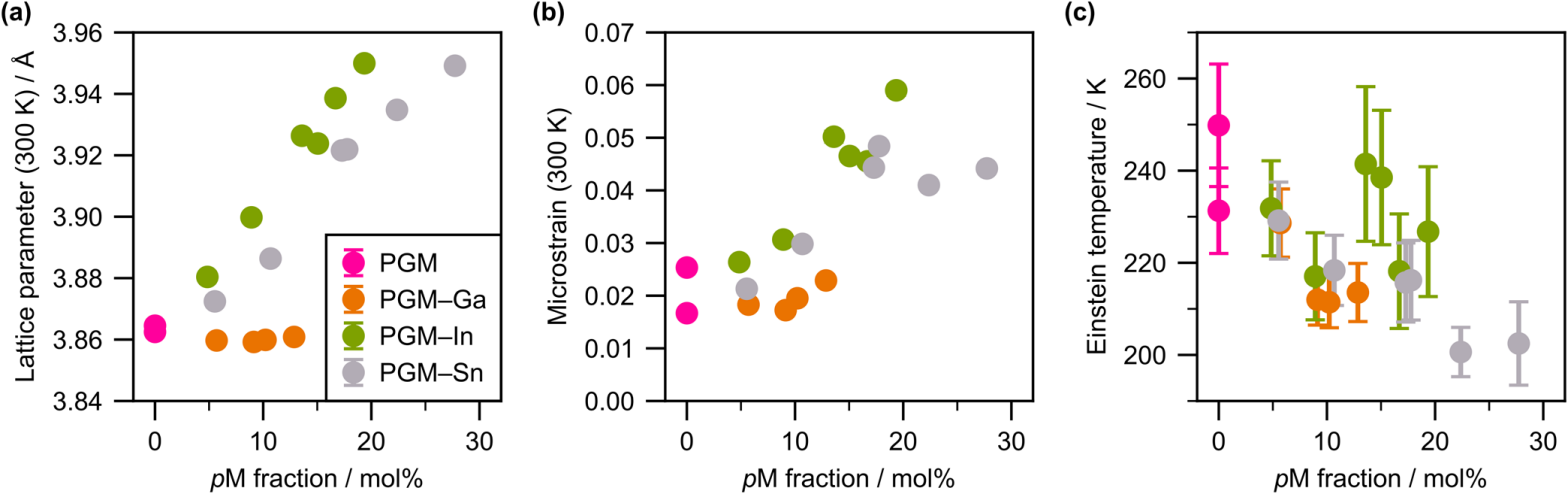
Compositional dependences of the (a) lattice parameters at 300 K, (b) microstrains at 300 K, and (c) Einstein temperatures. The compositions were the values determined by XRF.

To discuss the compositional dependence of the structures more quantitatively, we expressed the lattice parameters as linear combinations of composition-weighted atomic radii. The lattice parameters of the PGM MEA NPs well agreed with the value predicted based on the average atomic radii of PGMs in the monometals, as reported previously.^41^ The atomic radii of *p*Ms were estimated by linear regression (Figure S10 and Table S5, see Supporting Information Section S2.5 for details). The estimated values for Ga (1.354(6) Å), In (1.519(7) Å), and Sn (1.475(4) Å) were unignorably different from those in the corresponding monometals^29^ (1.22, 1.63, and 1.51 Å, respectively, in thermodynamically stable structures under ambient conditions). This result was attributed to the environments around the *p*Ms in the PGM–*p*M MEA NPs differing from those in the monometals, because of the differences in crystal structures and electronic interactions with PGMs.

### Element-Selective Structure Analyses by EXAFS

Although the above results indicate that the nature of the constituent elements and their interactions affect the alloy structures, this is only an indirect discussion based on the average structures. For instance, the estimation of the atomic radii assumes a linear compositional dependence, which might not always be the case.^42^ Moreover, the estimated atomic radii will be inaccurate if the composition of the alloy is inaccurate owing to the presence of surface oxides. Thus, EXAFS is a powerful tool for obtaining direct evidence of element-selective structures. X-ray absorption spectroscopy (XAS) measurements were performed at the SPring-8 BL01B1 beamline in transmission mode. The spectra of four samples synthesized using equimolar precursor ratios were acquired at the absorption edges of all constituent elements and seven temperatures from 8 to 373 K. The measurement temperatures were set low enough to avoid structural changes during the measurements and to acquire the spectra with large signal-to-noise ratios.

The difficulty in fitting the EXAFS of MEA NPs is mainly due to the large number of fitting parameters exceeding the information content of the experimental data.^19^ Although the information per spectrum is independent of the number of alloy components, the number of scattering paths required to describe the EXAFS oscillation increases proportionally to the number of components, adding at least four parameters per path: coordination number *N*, correction term of edge energy Δ*E*_0_, bond length *R*, and mean square relative displacement (MSRD) σ^2^. Therefore, for the EXAFS fitting of the MEA NPs, the number of parameters should be reduced by imposing constraints thereon. Some prior attempts at the EXAFS fitting of MEA NPs or bulk MEAs assumed that all paths shared a single *R* or σ^2^ value, neglecting structural variety.^20–22^ In other reports, the paths were grouped by the atomic numbers of the scattering atoms;^4,23–26^ however, this method cannot fully distinguish elements with similar atomic numbers. In addition, as the data at different absorption edges are usually fitted independently, the parameters describing the equivalent paths (element B observed from the absorption edge of element A and element A observed from element B) do not necessarily agree. To summarize, the currently reported methods omit the information on real structures and yield physically inconsistent structures.

To avoid these problems, we imposed constraints based on a physically reasonable and interpretable model and simultaneously fitted all spectra acquired at different absorption edges and temperatures. In other words, the number of parameters was reduced, whereas the amount of information was increased to overcome the above-mentioned issues. EXAFS oscillations arising from the first-nearest-neighbor pairs were fitted using four parameters (*N*, Δ*E*_0_, *R*, and σ^2^) per path by imposing following constraints. First, *N* and Δ*E*_0_ were considered to be independent of temperature, as no chemical and structural changes are expected at low temperatures (Figure 3a,b). Second, the consistency of *N* assigned to the equivalent paths at different edges was preserved (Figure 3a). Third, Δ*E*_0_ were set to be the same for the same absorption edge regardless of the scattering elements, as the variation in the chemical shifts within the alloy was assumed to be negligible (Figure 3b). Finally, *R* and σ^2^ were approximated as the sums of the contributions by the two elements involved in the bond (Figure 3c). For *R*, this model assumes that the bond length is the sum of the atomic radii, and for σ^2^, it means that MSRD is described as the sum of the mean square displacements (MSDs), approximating the atoms as independent oscillators or incorporating the correlation motion terms^43^ into MSDs. The above approximations neither omit structural information nor allow for structural inconsistencies (see Supporting Information Section S3.1 for details). Another advantage of this model is that result interpretation is straightforward as the individual contributions of each element to the alloy structures, namely atomic radii and MSDs, are explicitly estimated.

**Figure 3:**
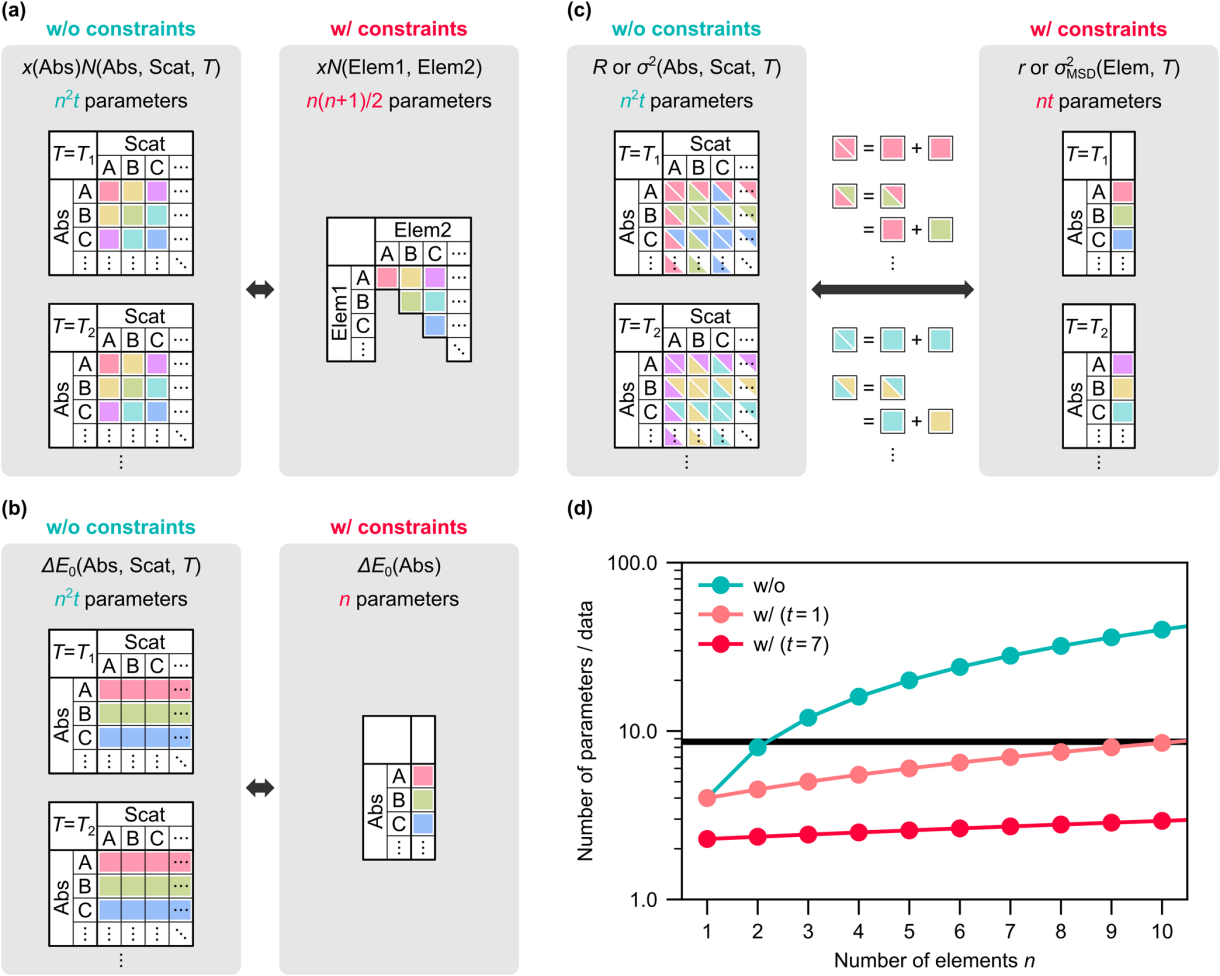
(a–c) Schematics illustrating the constraints imposed on (a) coordination number *N* (multiplied by compositions *x*), (b) correction term of edge energy Δ*E*_0_, and (c) bond length *R* and MSRD σ^2^. Tables in left and right boxes list the independent fitting parameters without and with the constraints, respectively; each cell represents a parameter in a one-to-one correspondence. The numbers of the parameters are given above the tables, assuming EXAFS data sets of *n*-element MEA NPs acquired at *t* temperatures. The parameter notations correspond to those in Supporting Information Section S3.1. Colors are used to describe the relationship between the parameters in the left and right boxes; in (a, b), those shown in the same color are set to have the same values. In (c), the parameters in the left box, shown in two colors, are assumed to be expressed as the sum of the two parameters shown in the corresponding colors in the right box. (d) Total numbers of independent parameters for MEAs with different *n*. Values are normalized by the numbers of data *nt*. Three cases are compared: (1) no constraints (w/o), (2) data at a single temperature analyzed with the constraints (w/(*t* = 1)), and (3) data at seven temperatures analyzed with the constraints (w/(*t* = 7)). Black horizontal line represents the upper limit when *k*-range = 10 Å^–1^ and *R*-range = 1.2 Å.

Figure 3d shows the total number of the independent fitting parameters in the alloys comprising different numbers of constituent elements without and with our constraints (see Supporting Information Section S3.2 for details). The upper limit^44^ is also shown, assuming typical analytical conditions. When no constraints are imposed, the number of parameters exceeds this limit, even for ternary alloys. In contrast, the number of independent parameters can be reduced to well below the limit by applying the constraints, enabling reliable analysis. Although our method works even when analyzing data acquired at a single temperature, the simultaneous fitting of data at several temperatures reduces the number of parameters relative to the limit more effectively, which is also advantageous for the evaluation of dynamic structures.

EXAFS fitting was performed with an originally developed Python script using the xraylarch (Larch^45^) library. Figure 4 shows the fitting results for the data of PGM–Sn MEA NPs acquired at 8 K as an example (the other results are listed in Figures S18–S20), revealing a high fit quality. The overall calculation required several tens of minutes to complete on a laptop (Table S9). The small computational cost is the advantage of our method over other prospective methods, such as the reverse Monte Carlo method^46,47^ or combinations with X-ray pair distribution function analyses.^48^

**Figure 4:**
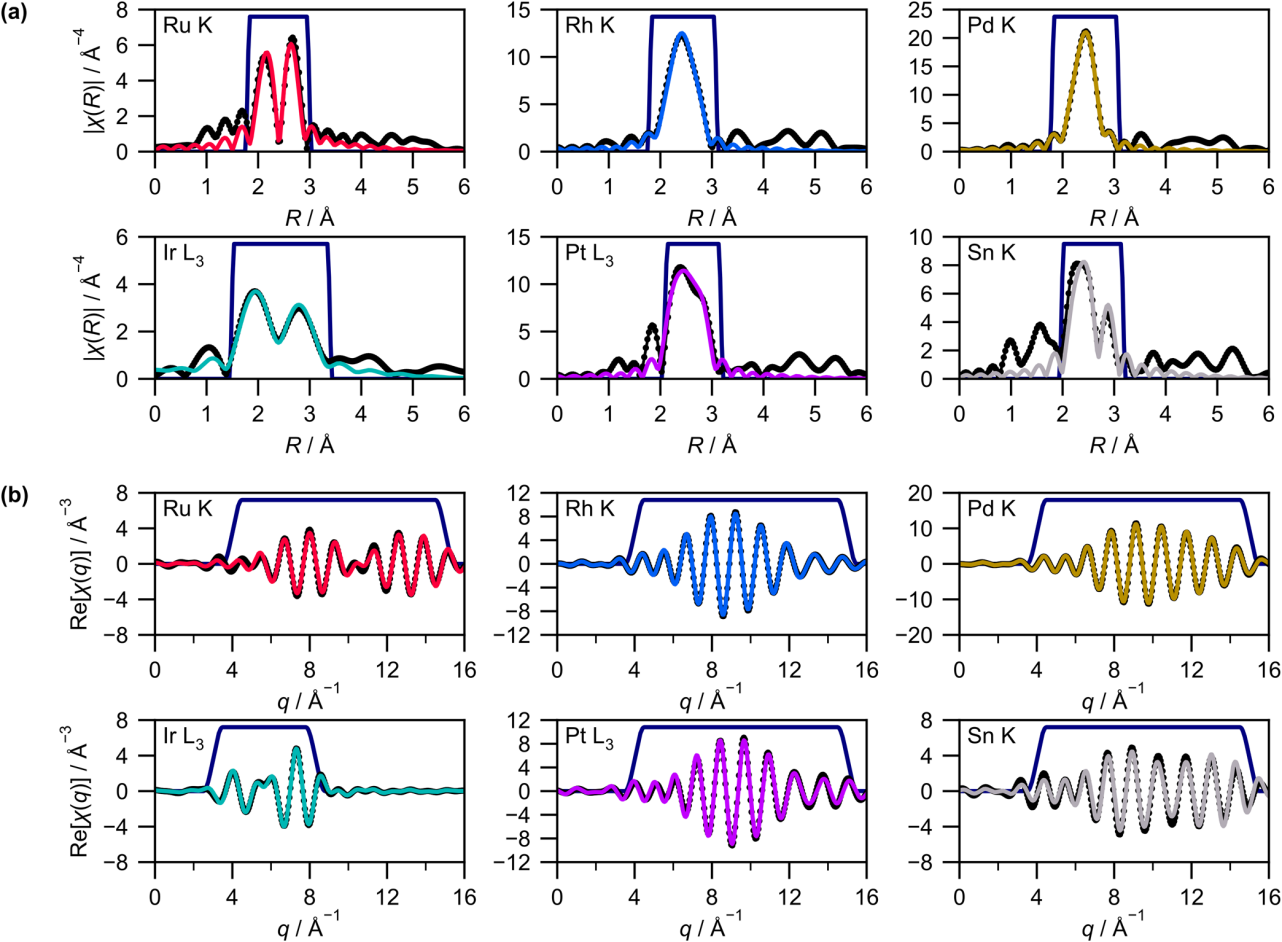
EXAFS fitting results of PGM–Sn MEA NPs synthesized using equimolar precursor ratios in (a) *R*-space (Fourier-transformed EXAFS) and (b) *q*-space (reverse Fourier-transformed EXAFS). Data acquired at 8 K are selected as examples. Black dots represent the experimental data, while the colored lines represent the fitting curves. Navy lines represent Fourier transform windows.

Below, the element dependences of static and dynamic local structures are discussed based on the estimated atomic radii and MSDs, respectively. Figure 5a summarizes the estimated atomic radii of the elements in each sample. As for the MSDs, attention should be paid to the correlation with *N*. Given that both *N* and σ^2^ influence the EXAFS amplitude, the estimation of these parameters often suffers from correlation issues. However, the temperature-dependent MSDs have almost the same offsets due to the correlation, and the temperature dependence is therefore not significantly affected (Figure S25, see Supporting Information Section S3.7 for details). Thus, the Einstein temperatures were derived by fitting the temperature dependences of the MSDs to the Einstein model^14^ (Figures S21–S24). The Einstein model was selected instead of the Debye model because of the compatibility of the former with the concept of resolving element-dependent local dynamic structures. For the same reason, the MSDs (one-atom oscillations) were fitted to the Einstein model, although MSRDs are commonly fitted to the Einstein model assuming an ensemble of two-atom oscillators. Although our approach oversimplifies the real samples because of inadequate treatment of correlated motions, the estimated Einstein temperatures were confirmed to be appropriate as an indicator of lattice hardness (Figures S28b and S29b, see Supporting Information Section S3.9 for details).^14,49^ Figure 5b summarizes the estimated Einstein temperatures. Both the atomic radii and Einstein temperatures reflect the trend in the monometals (Tables S4, S14, and S18). For example, the atomic radii increase and the Einstein temperatures decrease with the increasing number of the PGM valence electrons upon going from Ru to Pd or from Ir to Pt, which can be ascribed to the concomitant increase in the occupancies of the antibonding bands. In addition, In and Sn have larger atomic radii than PGMs, and the Einstein temperatures of *p*Ms are lower than those of PGMs. However, quantitatively, these results cannot be solely ascribed to the intrinsic nature of the elements. According to the XRD data acquired for the monometals (Table S4) and literature values (Table S18), the atomic radii of *p*Ms in the corresponding monometals (1.22, 1.63, and 1.51 Å for Ga, In, and Sn, respectively, in the thermodynamically stable structures) were significantly different from those of PGMs in the monometals (1.33–1.39 Å). However, the atomic radii of Ga, In, and Sn in the MEA NPs estimated from EXAFS data (1.290(7), 1.423(5), and 1.435(8) Å, respectively) were substantially closer to the PGM values. Also, the Einstein temperatures of monometallic In and Sn estimated by XRD (114(1) and 91.5(5) K, respectively) were distinctively lower than the value of Pt (149(1) K), the lowest among the PGMs. However, the Einstein temperatures of Ga, In, and Sn in the MEA NPs estimated from EXAFS data (186(15), 194(15), and 172(18) K, respectively) were comparable with the values of Pt (178(2) K in monometallic Pt, 178–212 K in the MEA NPs). Note that the Einstein temperatures estimated using XRD and EXAFS analysis are incompatible because the correlation motion terms are taken into MSDs in our EXAFS analysis, whereas the values are expected to have a linear relationship^49^ (Figure S29c, see Supporting Information Section S3.9 for details), as experimentally supported by the results of the XRD and EXAFS analyses of the monometallic PGMs (Figure S26). In contrast to *p*Ms, neither the atomic radii nor Einstein temperatures of the PGMs in the MEA NPs and corresponding monometals were distinctively different (Figure 5). The interactions among the PGMs with similar electronic properties were assumed to hardly affect the structures, whereas the interactions between PGMs and *p*Ms with contrasting properties were expected to have a stronger influence on the structures. Considering the PGM-rich composition of the MEA NPs, the fact that the PGMs retain their local structures, whereas the local structures around the *p*Ms are largely modulated in the alloys, is reasonable.

**Figure 5:**
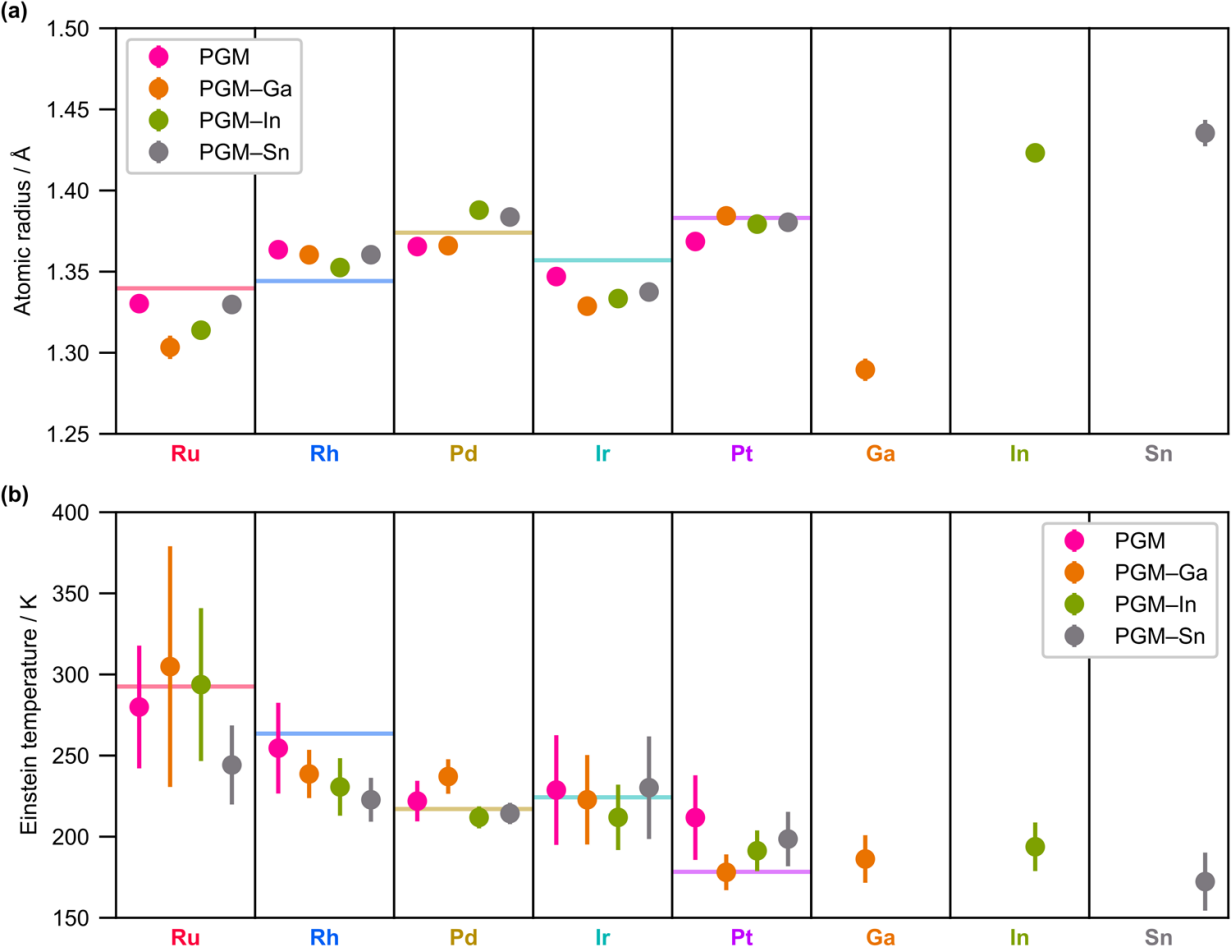
Estimated element-dependent (a) atomic radii and (b) Einstein temperatures in the MEA NPs synthesized using equimolar precursor ratios. Different panels correspond to different elements, and differently colored markers in the same panel refer to the same element in different MEA NPs. Horizontal lines represent the values in the monometallic PGMs estimated from EXAFS data (Table S14).

### *Ab Initio* Calculations for Resolving Elemental Interactions

To resolve the influence of individual elemental interactions within MEA NPs on the alloy structures, we performed density functional theory (DFT) calculations. Direct modeling of MEAs is computationally impractical and the interpretation of the results becomes complicated, so we approximated the systems by using a series of L1_2_ binary alloys formulated as X_3_Y, where X and Y are among the eight elements in the MEA NPs (Ru, Rh, Pd, Ir, Pt, Ga, In, and Sn). The lattice parameters of the geometry-optimized structures in PGM-rich alloys (X = PGM) could be well described using a linear combination weighted by composition. The atomic radius of each element was estimated using linear regression (Figures S32 and S33 and Table S17). Remarkably, the atomic radii of *p*Ms in PGM-rich alloys (1.376(3), 1.533(3), and 1.537(3) Å for Ga, In, and Sn, respectively) were considerably smaller than those in the corresponding monometallic *p*Ms (1.497, 1.687, and 1.705 Å, respectively), while the atomic radii of PGMs in the monometals and alloys were in good agreement (Table S17). These findings help explain the behavior of *p*Ms in the PGM–*p*M MEA NPs estimated by the XRD and EXAFS analyses. The Einstein temperatures around a certain element were evaluated by calculating the potential energy curves of one-atom displacements (Figures S34 and S35, see Supporting Information Section S4.2 for details). Although the Einstein temperatures in PGM–PGM alloys and *p*M–*p*M alloys were similar to those in the respective monometals, the Einstein temperatures around the *p*Ms increased, and those around the PGMs decreased in the alloys of a PGM and a *p*M. Specifically, the Einstein temperatures around the *p*Ms in PGM-rich alloys (174–254 K) markedly exceeded those in the monometallic *p*Ms (125.2(2), 96(1), and 87.3(1) K for Ga, In, and Sn, respectively) and were comparable with those in the monometallic PGMs (173–254 K). These results explain the trends estimated using EXAFS. Furthermore, to clarify the origin of the structural modulation due to the PGM–*p*M interaction in terms of electronic structures, we calculated the densities of states (DOSs) of the alloys using DFT. The comparison of the local projected DOSs of *p*Ms in the PGM–*p*M alloys and the monometallic fcc *p*Ms revealed additional peaks at the bottom of the valence bands in the alloys, which were absent in the monometals (Figure S36). This suggests the presence of bonding interactions between PGMs and *p*Ms. The bonding energy gain from these interactions likely explains the smaller atomic radii and higher Einstein temperatures of the *p*Ms in the PGM–*p*M MEA NPs.

## Perspectives

At present, our analysis suffers from an experimental issue: a spectral overlap of absorption edges with proximate energies, which is inevitable in multielement systems. For example, Ir and Pt L_3_-edges are only 350 eV apart. The *k*-ranges of Ir L_3_-edge were shortened because of interruption by Pt L_3_-edge jumps. It significantly reduced the structural information around Ir atoms. Moreover, the Pt L_3_-edge spectra were affected by Ir L_3_ EXAFS. Peaks at 1.5–2.0 Å found in the Pt L_3_ FT-EXAFS of the MEA NPs (Figures 4a and S18a–20a) are artifacts due to the overlap (Figures S30 and S31a). Although we verified that the overlap issue causes only small errors in the estimation of atomic radii and Einstein temperatures, which supports the reliability of the discussion above, the estimation of coordination numbers and static strains might be susceptible to the issue because of the correlation between *N* and σ^2^ (Figure S31b and Tables S15 and S16, see Supporting Information Section S3.10 for details). We are expecting that the accuracy of our analysis can be improved by experimental modification to avoid the overlaps: such as use of K-edge EXAFS^50^ or “range-extended EXAFS” using high-energy resolution fluorescence mode.^51–54^ In addition to the experimental approach, the modification in data analysis is desired. The model constraints can be modified depending on preliminary knowledge of the system including computational structure predictions. The improvements of the models can contribute not only to accuracy improvement but also to extending our method to a broader range of targets such as multielement oxides^55^ or other ceramics.^56^

There are several benefits expected to be brought by the improvements. Among those is elemental arrangement analysis based on the coordination numbers. The coordination numbers estimated by EXAFS analyses should be informative of homogeneity or short-range order (SRO) in the alloys,^19^ which is a critical factor for understanding and controlling their properties.^10,57^ Although the estimated coordination numbers in the present results were not reliable enough to make quantitative discussion as mentioned above, the qualitative trend (Tables S10–S13) seems reasonable considering the elemental distribution found in the EDX maps (Figures 1a and S3). For instance, the EDX maps showed that Ru and Ir tended to be concentrated around the NP surface, whereas Pd tended to be concentrated at the core. Consistently, the coordination numbers estimated by EXAFS analyses showed that Ru–Pd and Pd–Ir pairs were avoided, whereas Ru–Ir pairs were preferred. These trends are also reasonable in terms of the affinity between the elements, represented as binary phase diagrams^58–60^ and thermally driven dealloying of Pd from Ir–Pd–Ru alloy.^61^ We also evaluated the affinity as the formation energies of the binary alloys (Figure S37); Ru–Pd and Pd–Ir pairs are unstable while a Ru–Ir pair is stable. Although random elemental distributions tend to be stabilized in MEAs because of entropy effect,^2,3,62^ the competing contribution of enthalpy would lead to short-range order or heterogeneous distributions. To achieve more quantitative discussion by suppressing the *N* – σ^2^ correlation issue, one of the possible analytical modifications is to impose constraints on the sum of coordination numbers (for example, 12 in fcc). Although this assumption is valid for monophasic bulk MEAs, its application to MEA NPs is challenged by the unignorable contributions from surfaces, defects, and oxide byproducts. Given that the homogeneity and SRO of the MEA NPs depend on the synthesis conditions or working environment of the material, their computational prediction is difficult. Therefore, experimental methods for characterizing the homogeneity or SRO of MEA NPs are indispensable, and the results can be used as the initial structures for computational discussions. In addition, it would be useful for investigating the structurally ordered MEA NPs, also known as high-entropy intermetallic NPs, which have recently emerged as a new class of high-entropy materials.^63–65^ Besides the coordination numbers, the accuracy improvements would enable discussion on the small changes which could not be distinguished in the present results, such as structural influence on PGMs by *p*Ms or surface softening effects unique to nanoparticles.^66–68^

## Conclusions

We proposed the quantitative analysis method for investigating element-selective structures in MEA NPs using EXAFS. The physically consistent and interpretable analyses were achieved using a combination of the simultaneous fitting of multiple data and reduction of the fitting parameters based on the model equations. This method was applied to the MEA NPs composed of PGMs and *p*Ms, which have contrasting structural and electronic properties. The estimated element-dependent atomic radii and Einstein temperatures reflected the intrinsic nature of the elements but were not solely ascribed to them. The electronic interactions between *p*Ms and PGMs led to the distinctively shrunken and hardened lattices around the *p*Ms compared with those in the corresponding monometals. As demonstrated in this example, our method is a simple but powerful approach for unraveling the structural complexity of multielement materials arising from the coexistence of elements of various natures and their interactions. This technique is expected to facilitate the experimental investigation of these structurally complicated nanomaterials, which have usually been discussed computationally, leading to a precise understanding of the structure–property relationships in real systems.

## Experimental Section

### Synthesis of MEA NPs

MEA NPs were synthesized by injecting an oleylamine solution of metal precursors into a heated oleylamine solution of d-glucose. First, the metal precursor solution was prepared. The metal precursors were triruthenium dodecacarbonyl (Ru_3_(CO)_12_, Sigma-Aldrich), rhodium­(III) chloride trihydrate (RhCl_3_·3H_2_O, Wako, Japan), palladium­(II) bis­(acetylacetonate) (Pd­(C_5_H_7_O_2_)_2_, Wako, Japan), iridium acetate (Ir­(CH_3_COO)_n_, Wako, Japan), platinum­(II) bis­(acetylacetonate) (Pt­(C_5_H_7_O_2_)_2_, Wako, Japan), gallium­(III) chloride (GaCl_3_, Wako, Japan), indium­(III) chloride tetrahydrate (InCl_3_·4H_2_O, Nacalai Tesque, Japan), and tin­(IV) chloride pentahydrate (SnCl_4_·5H_2_O, Wako, Japan). The precursors (0.60 mmol in total) were dissolved in oleylamine (15 mL; Wako, Japan). Second, oleylamine (75 mL) was placed in a three-necked flask with d-glucose (3.0 mmol; Wako, Japan) to help reduction of metal precursors. This solution was evacuated, purged with Ar several times, and heated to 290 °C under a flow of Ar. Subsequently, the metal precursor solution was injected into the heated solution at a rate of 0.50 mL/min. After the injection was completed, the solution was heated to 310 °C, kept at this temperature for 1 h, and cooled to room temperature. The obtained, black solution was mixed with moderate amounts of hexane, ethyl acetate, and acetone and centrifuged to precipitate the NPs as black powders.

Fixed equimolar PGM precursor ratios were used, whereas the *p*M fractions were varied from 0 to 25%. Eighteen samples were synthesized using different precursor ratios. The PGM–*p*M MEA NPs were named as “PGM–*p*Mxx”, where “*p*M” is Ga, In, or Sn, and “xx” is the nominal molar fraction of the *p*M. Samples with equimolar precursor ratios were synthesized in two different batches: the first of which (suffixed as _1; PGM_1, PGM–Ga17_1, PGM–In17_1, and PGM–Sn17_1) was used for EDX mapping and XPS, and the other (suffixed as _2; PGM_2, PGM–Ga17_2, PGM–In17_2, and PGM–Sn17_2) was used for XAS (XANES and EXAFS). The sample names and precursor ratios are listed in Table S1.

### XRF Spectroscopy

The compositions of 18 samples were determined by XRF spectroscopy using a ZSX Primus IV (Rigaku, Japan) and are summarized in Table S1.

### TEM

The TEM images of 18 samples were acquired using an HT7700 (Hitachi, Japan) at an acceleration voltage of 100 kV. Figure S1 shows the TEM images of different MEA NPs.

### EDX Mapping

The EDX elemental maps of four samples (PGM_1, PGM–Ga17_1, PGM–In17_1, and PGM–Sn17_1) were acquired using a JEM-ARM200CF instrument (JEOL, Japan) equipped with an EDX detector. The acceleration voltage was set to 120 kV. Figure S3 shows the EDX maps of the PGM MEA NPs (PGM_1) and Figure 1a shows those of the PGM–*p*M MEA NPs (PGM–Ga17_1, PGM–In17_1, and PGM–Sn17_1).

### XPS

The XPS spectra of three samples (PGM–Ga17_1, PGM–In17_1, and PGM–Sn17_1) were acquired using an ESCA-3400 (SHIMADZU, Japan) with an Al Kα radiation source (1486.61 eV). The measurements were performed before and after Ar-ion sputtering (20 mA, 2.0 kV, 1 min) to investigate the chemical states of the surface and core parts of the NPs. The spectra of *p*M core levels (Ga 2*p*, In 3*d*, and Sn 4*d*) are shown in Figure 1b.

### XRD

XRD measurements were performed at the SPring-8 BL13XU beamline using an incident X-ray energy of 60 keV. The samples were placed in borosilicate glass capillaries (W. Müller GmbH, Germany). The XRD profiles were acquired at five temperatures (105, 150, 200, 250, and 300 K). The sample temperature was controlled by spraying temperature-controlled N_2_ gas. Figure S6 shows the XRD profiles of all samples at 105 K. Figure S7 shows the temperature-dependent XRD profile of PGM–Ga17_2 as an example.

### XAS

XAS measurements were performed at the SPring-8 BL01B1 beamline in transmission mode. The incident X-ray was monochromated using a Si (311) double-crystal monochromator. The samples were mixed with boron nitride (Kojundo Chemical Laboratory Co., Ltd., Japan) to prepare pellets with moderate edge jumps. The measurements for XANES analyses were performed on 14 samples (excluding PGM_1, PGM–Ga17_1, PGM–In17_1, and PGM–Sn17_1) at room temperature. The monochromator was swept in the quick scan mode (QXAFS). The XANES spectra of selected samples and references are shown in Figure S4.

The variable-temperature measurements for EXAFS analyses were performed at seven temperatures (8, 50, 100, 150, 200, 300, and 373 K) for PGM_2, PGM–Ga17_2, PGM–In17_2, and PGM–Sn17_2. The measurements at low temperatures (≤300 K) were performed using a cryostat, whereas those at a high temperature (373 K) were performed under He gas flow using an *in*-*situ* cell at the beamline. The monochromator was swept in the step scan mode during the cryostat operation (≤200 K), while the other measurements (300 and 373 K) were performed in the QXAFS mode.

### DFT Calculations

DFT calculations were carried out using the projector augmented wave method as implemented in the Vienna Ab Initio Simulation Package code^69,70^ version 5.4.4. The Perdew–Burke–Ernzerhof functional^71^ within the framework of the generalized gradient approximation was employed to describe the exchange–correlation energies. All the calculations were performed without spin polarization. The target systems included a series of binary L1_2_ alloys and fcc monometals composed of PGMs and *p*Ms. Geometry optimization and DOS calculation were conducted using 4-atom unit cells. The Einstein temperatures were estimated by displacing a single atom within 2 × 2 × 2 supercells and fitting the potential curve under a harmonic approximation (see Supporting Information Section S4.2 for details). A Gaussian smearing scheme with ISMEAR = 1 and SIGMA = 0.1 eV was used for electronic structure calculations. The *k*-point mesh was 20 × 20 × 20 for the 4-atom unit cells and 10 × 10 × 10 for the 32-atom supercells, ensuring convergence of total energy within 1.5 meV per atom. The plane-wave basis set cutoff energy was 600 eV, which was chosen to guarantee sufficient accuracy in the total energy calculations. Convergence criteria were 10^–8^ eV for electronic steps and 10^–7^ eV for ionic steps per 4-atom unit cell.

## Supplementary Material



## Data Availability

All data are available in the main text or Supporting Information, or from the corresponding author upon reasonable request. The experimental data, codes, and results of EXAFS analyses will be made publicly available in a GitHub repository (http://github.com/masasnakamura/2025_ACSNanosciAu_EXAFS) upon acceptance of the manuscript.

## Notes

General characterizations; XRD analysis; EXAFS analysis; and computational details (PDF)

## ○

School of Materials Science and Engineering, College of Engineering, Nanyang Technological University, 50 Nanyang Avenue, North Spine, Block N4.1–02–16, 639798 Singapore
