# Supplementary material for: Unraveling Element-Selective Local Structures in Multielement Alloy Nanoparticles with EXAFS
Source: ACS Nanosci Au. 2025 Apr 30;5(3):196–207. doi: 10.1021/acsnanoscienceau.5c00013 (PMC12183595; doi:10.1021/acsnanoscienceau.5c00013)
Supplement: Supplementary file 1 [file ng5c00013_si_001.pdf]

Supporting Information

Unraveling Element-Selective Local Structures in Multielement Alloy Nanoparticles with EXAFS

Masashi Nakamura,*[1] Dongshuang Wu,*[1] Megumi Mukoyoshi,*[1] Kohei Kusada,[1,2] Hiroyuki Hayashi,[3] Takaaki Toriyama,[4] Tomokazu Yamamoto,[4] Yasukazu Murakami,[4,5] Hirotaka Ashitani,[6] Shogo Kawaguchi,[6] Toshiaki Ina,[6] Osami Sakata,[6] Yoshiki Kubota,[7] Isao Tanaka,[3] and Hiroshi Kitagawa*[1]

1. Division of Chemistry, Graduate School of Science, Kyoto University, Kitashirakawa-Oiwakecho, Sakyo-ku, Kyoto 606-8502, Japan
2. The HAKUBI Center for Advanced Research, Kyoto University, Kitashirakawa-Oiwakecho, Sakyo-ku, Kyoto 606-8502, Japan
3. Department of Materials Science and Engineering, Graduate School of Engineering, Kyoto University, Yoshida-Honmachi, Sakyo-ku, Kyoto 606-8501, Japan
4. The Ultramicroscopy Research Center, Kyushu University, 744 Motooka, Nishi-ku, Fukuoka 819-0395, Japan
5. Department of Applied Quantum Physics and Nuclear Engineering, Graduate School of Engineering, Kyushu University, 744 Motooka, Nishi-ku, Fukuoka 819-0395, Japan
6. Japan Synchrotron Radiation Research Institute (JASRI), SPring-8, 1-1-1 Kouto, Sayo-cho, Sayo-gun, Hyogo 679-5198, Japan
7. Department of Physics, Graduate School of Science, Osaka Metropolitan University, 1-1 Gakuen-cho, Naka-ku, Sakai, Osaka 599-8531, Japan

E-mails

Masashi Nakamura : nakamura.masashi.72w@st.kyoto-u.ac.jp

Dongshuang Wu : dongshuang.wu@ntu.edu.sg

Megumi Mukoyoshi : mukoyoshi@ssc.kuchem.kyoto-u.ac.jp

Hiroshi Kitagawa : kitagawa@kuchem.kyoto-u.ac.jp

§1. General Characterizations of MEA NPs

§1.1. XRF Spectroscopy

Table S1 Nominal and XRF-determined compositions (mol%) of the MEA NPs.

Sample ID		Ru	Rh	Pd	Ir	Pt	Ga	In	Sn
**PGM_1**	Nominal	20.0	20.0	20.0	20.0	20.0	-	-	-
	XRF	20.7	18.7	21.3	20.3	19.0	-	-	-
**PGM_2**	Nominal	20.0	20.0	20.0	20.0	20.0	-	-	-
	XRF	19.9	20.1	21.9	19.5	18.7	-	-	-
**PGM–Ga10**	Nominal	18.0	18.0	18.0	18.0	18.0	10.0	-	-
	XRF	19.8	17.9	21.7	15.6	19.4	5.7	-	-
**PGM–Ga17_1**	Nominal	16.7	16.7	16.7	16.7	16.7	16.7	-	-
	XRF	16.0	18.1	20.4	17.8	17.4	10.2	-	-
**PGM–Ga17_2**	Nominal	16.7	16.7	16.7	16.7	16.7	16.7	-	-
	XRF	17.8	20.6	21.2	16.0	15.3	9.1	-	-
**PGM–Ga25**	Nominal	15.0	15.0	15.0	15.0	15.0	25.0	-	-
	XRF	15.8	17.7	19.9	17.0	16.8	12.9	-	-
**PGM–In05**	Nominal	19.0	19.0	19.0	19.0	19.0	-	5.0	-
	XRF	19.4	18.9	19.4	19.0	18.5	-	4.8	-
**PGM–In10**	Nominal	18.0	18.0	18.0	18.0	18.0	-	10.0	-
	XRF	17.1	17.0	18.0	19.7	19.3	-	8.9	-
**PGM–In17_1**	Nominal	16.7	16.7	16.7	16.7	16.7	-	16.7	-
	XRF	16.1	17.8	17.3	16.6	17.1	-	15.1	-
**PGM–In17_2**	Nominal	16.7	16.7	16.7	16.7	16.7	-	16.7	-
	XRF	16.6	17.2	17.7	17.9	16.9	-	13.6	-
**PGM–In20**	Nominal	16.0	16.0	16.0	16.0	16.0	-	20.0	-
	XRF	15.7	16.4	16.7	17.7	16.7	-	16.7	-
**PGM–In25**	Nominal	15.0	15.0	15.0	15.0	15.0	-	25.0	-
	XRF	15.5	16.2	16.1	16.7	16.1	-	19.4	-
**PGM–Sn05**	Nominal	19.0	19.0	19.0	19.0	19.0	-	-	5.0
	XRF	19.8	19.0	20.2	17.9	17.6	-	-	5.5
**PGM–Sn10**	Nominal	18.0	18.0	18.0	18.0	18.0	-	-	10.0
	XRF	18.4	17.9	19.0	17.4	16.6	-	-	10.7
**PGM–Sn17_1**	Nominal	16.7	16.7	16.7	16.7	16.7	-	-	16.7
	XRF	17.1	16.2	18.0	16.1	14.9	-	-	17.8
**PGM–Sn17_2**	Nominal	16.7	16.7	16.7	16.7	16.7	-	-	16.7
	XRF	16.6	17.7	17.5	15.8	15.2	-	-	17.3
**PGM–Sn20**	Nominal	16.0	16.0	16.0	16.0	16.0	-	-	20.0
	XRF	15.9	16.2	17.5	14.1	13.9	-	-	22.4
**PGM–Sn25**	Nominal	15.0	15.0	15.0	15.0	15.0	-	-	25.0
	XRF	16.4	15.1	17.6	13.1	10.0	-	-	27.7

§1.2. TEM

Figure S1 shows the TEM images of different samples (several views were acquired for each sample, although only one view is shown). Particle sizes were measured using the ImageJ software.[1] To obtain statistically reliable values, more than 300 NPs were counted. **Figure S2** shows the size distributions based on the number of particles, not on the volume.

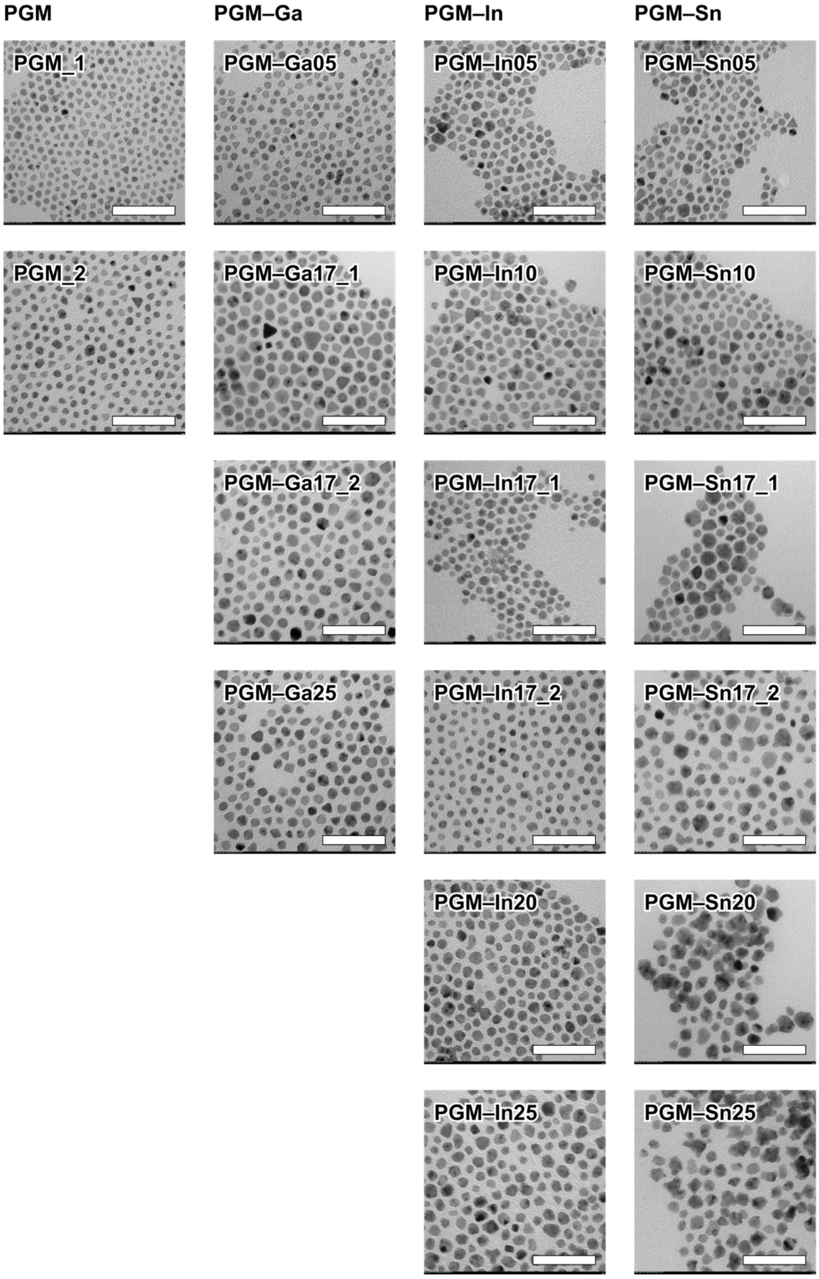

Figure S1 TEM images of the MEA NPs at the same magnification. The scale bars indicate 50 nm.

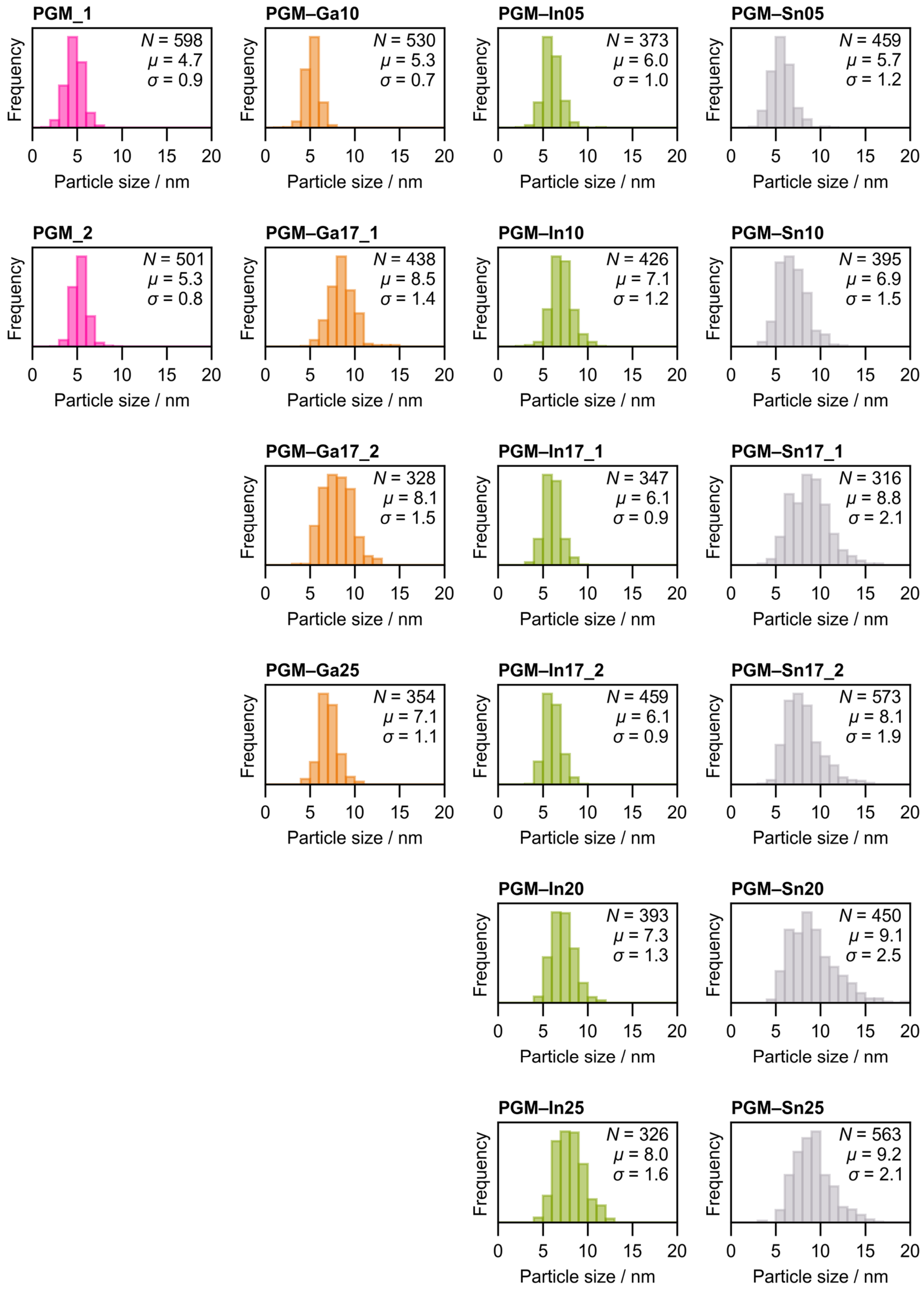

Figure S2 Particle size distributions of the MEA NPs determined by TEM image analysis (N: sample population, μ: mean value (nm), σ: standard deviation (nm)).

§1.3. EDX Mapping

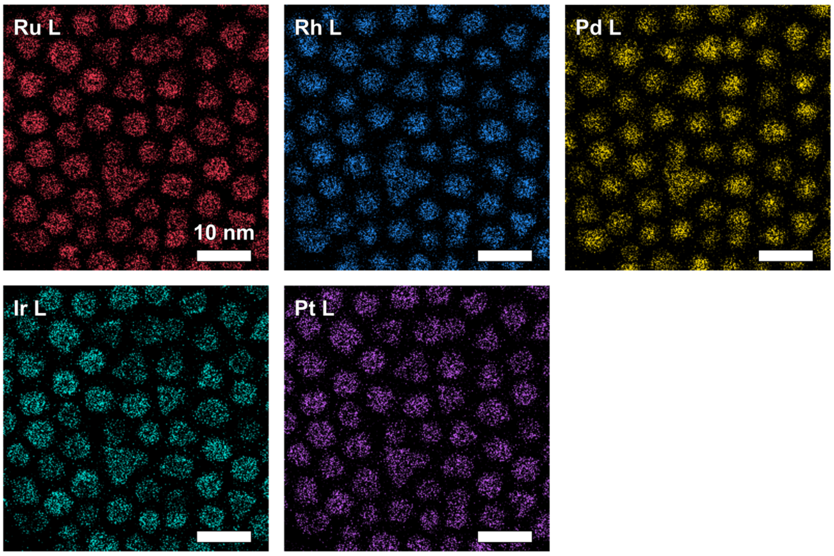

Figure S3 Elemental maps of PGM_1 obtained by STEM–EDX.

§1.4. XPS

The XPS spectra were fitted as follows. The backgrounds were determined using the Shirley method or linear interpolation, and peak deconvolution was then performed. Peak shapes were described using a pseudo-Voigt function. The spectra before and after the Ar-ion sputtering were simultaneously fitted assuming the same peak positions, widths, and shapes. The doublet separation energies due to spin–orbit coupling were fixed at typical literature values.

Ga 2*p*, In 3*d*, and Sn 4*d* were selected as targets with large photo-ionization cross-sections while avoiding spectral overlap. Fitting was performed assuming two components, metal and oxide. The experimental data and fitting results are shown in **Figure 1b** and **Table S2**. The fraction of the metal component increased after the sputtering, which indicated the presence of *p*M oxides on the NP surface and metallic *p*Ms inside the NPs.

Table S2 (**a**) The estimated binding energies (eV) of the *p*M core levels in different samples. Two bottom rows show values previously reported for monometals and oxides in their common valence states.[2] (**b**) Fractions (mol%) of metal components before and after the Ar-ion sputtering estimated based on peak areas (residuals are oxide components).

(a)

Sample<br>Orbital		PGM–Ga17_1<br>Ga $2p_{3/2}$	PGM–In17_1<br>In $3d_{5/2}$	PGM–Sn17_1<br>Sn $4d_{5/2}$
Sample	Metal	1116.928(6)	443.786(3)	24.434(5)
	Oxide	1118.76(2)	444.91(3)	25.77(5)
Ref.	Metal	1117.0	443.8	24.0
	Oxide<br>(valence)	1117.5<br>(III)	444.7<br>(III)	25.9<br>(IV)

(b)

Sample<br>Orbital	PGM–Ga17_1<br>Ga $2p_{3/2}$	PGM–In17_1<br>In $3d_{5/2}$	PGM–Sn17_1<br>Sn $4d_{5/2}$
Before	30(1)	52(5)	39(2)
After	64(1)	82(1)	75(1)

§1.5. XANES

The XANES spectra were analyzed using the Demeter Athena software.[3] The normalized spectra of four samples (PGM_2, PGM–Ga17_2, PGM–In17_2, and PGM–Sn17_2) at *p*M K-edges and PGM K or L_3-edges are shown in **Figure 1c** and **Figure S4**, respectively. To compare the chemical states of PGMs in the different samples, the representative values of the spectra were extracted. Regarding the K-edges, the absorption energies E_0 were determined as the energies at which the normalized μt equals 0.5 (a fraction of the edge jump). Regarding the L_3-edges, the peaktop intensities of the whiteline (WL) were compared. Monotonous changes in the spectral features with compositions were observed (**Figure S5**). Generally, negative chemical shifts and lower WL intensities indicate the electron-rich valence states. Therefore, the smaller E_0 values and WL intensities observed for the *p*M-rich samples were due to electron transfer from the electropositive *p*Ms to the electronegative PGMs. The violation of the trend observed for Ru would be due to the presence of an oxide component, as Ru is the most prone to oxidation among the PGMs. The results of XANES and Fourier-transformed EXAFS indicated that Ru was partially oxidized.

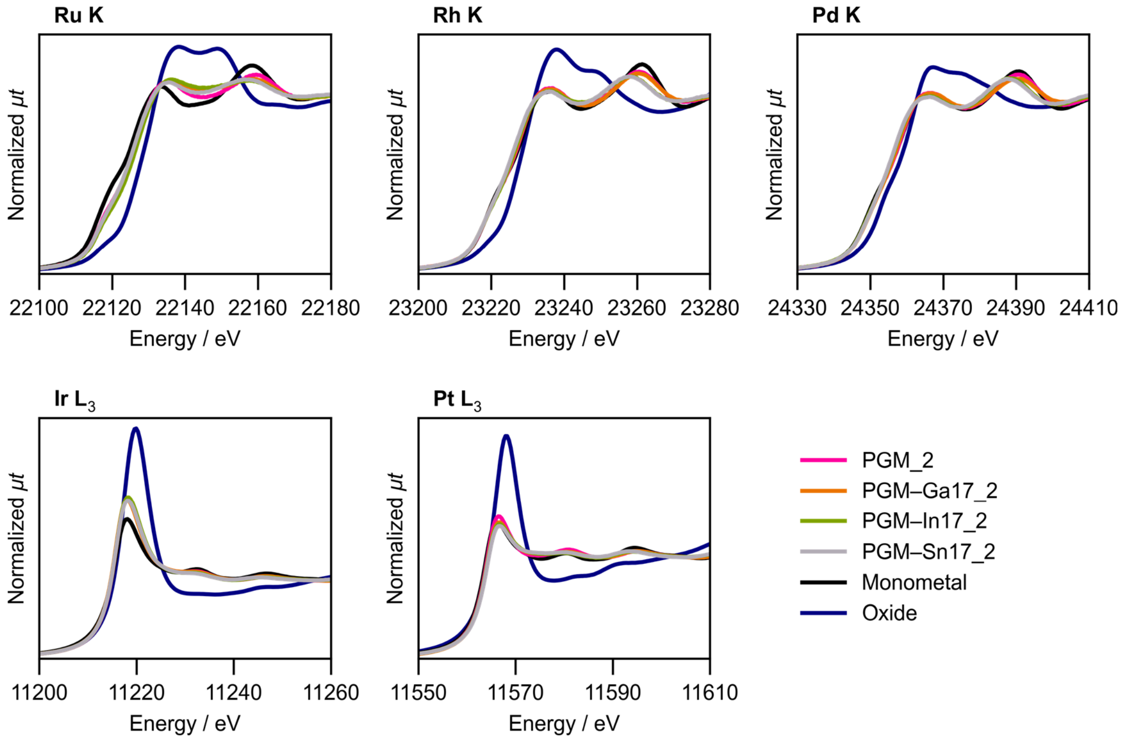

Figure S4 XANES spectra at the absorption edges of PGMs in the MEA NPs and references (monometals and common oxides). The K-edge spectra were measured for Ru, Rh, and Pd, and the L_3-edge spectra were measured for Ir and Pt. Reference oxides are RuO_2, Rh_2O_3, PdO, IrO_2, and PtO_2, respectively.

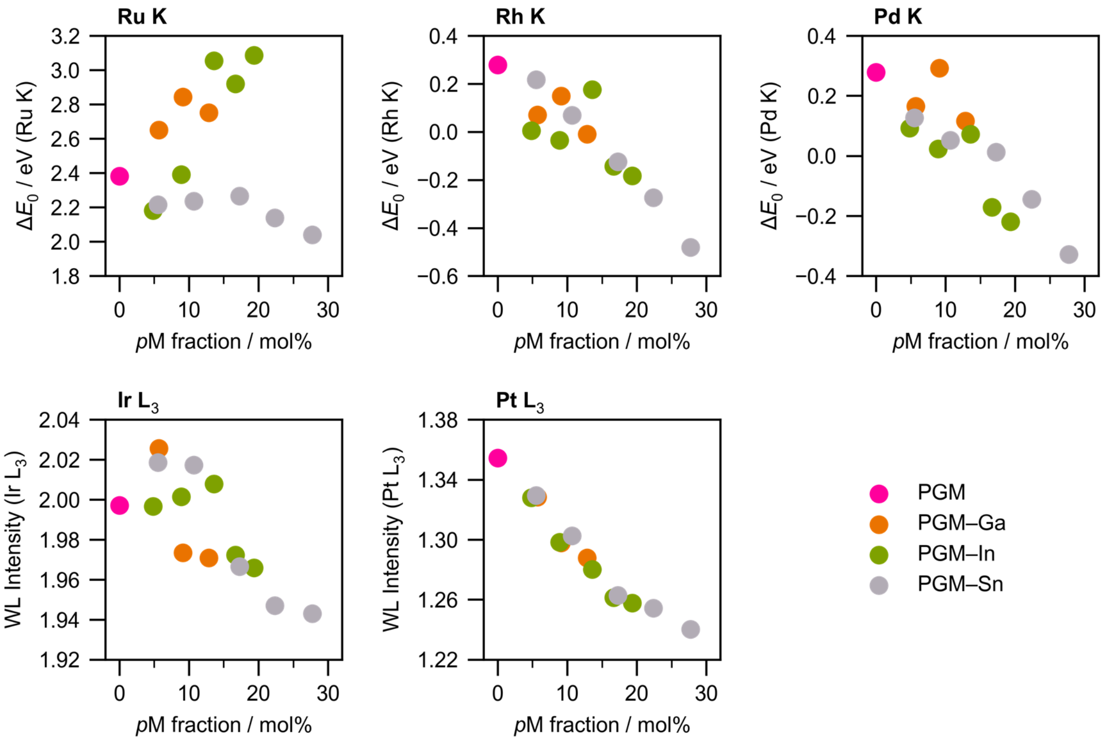

Figure S5 Composition (pM molar fraction) dependences of the XANES spectral features of PGMs in the MEA NPs: shift of absorption energies E_0 relative to the corresponding monometals at Ru, Rh, and Pd K-edges, and WL peaktop heights at Ir and Pt L_3-edges. The marker in different colors represent the MEAs composed of different combinations of elements.

§2. Average Structure Analysis by XRD

§2.1. Experimental Data

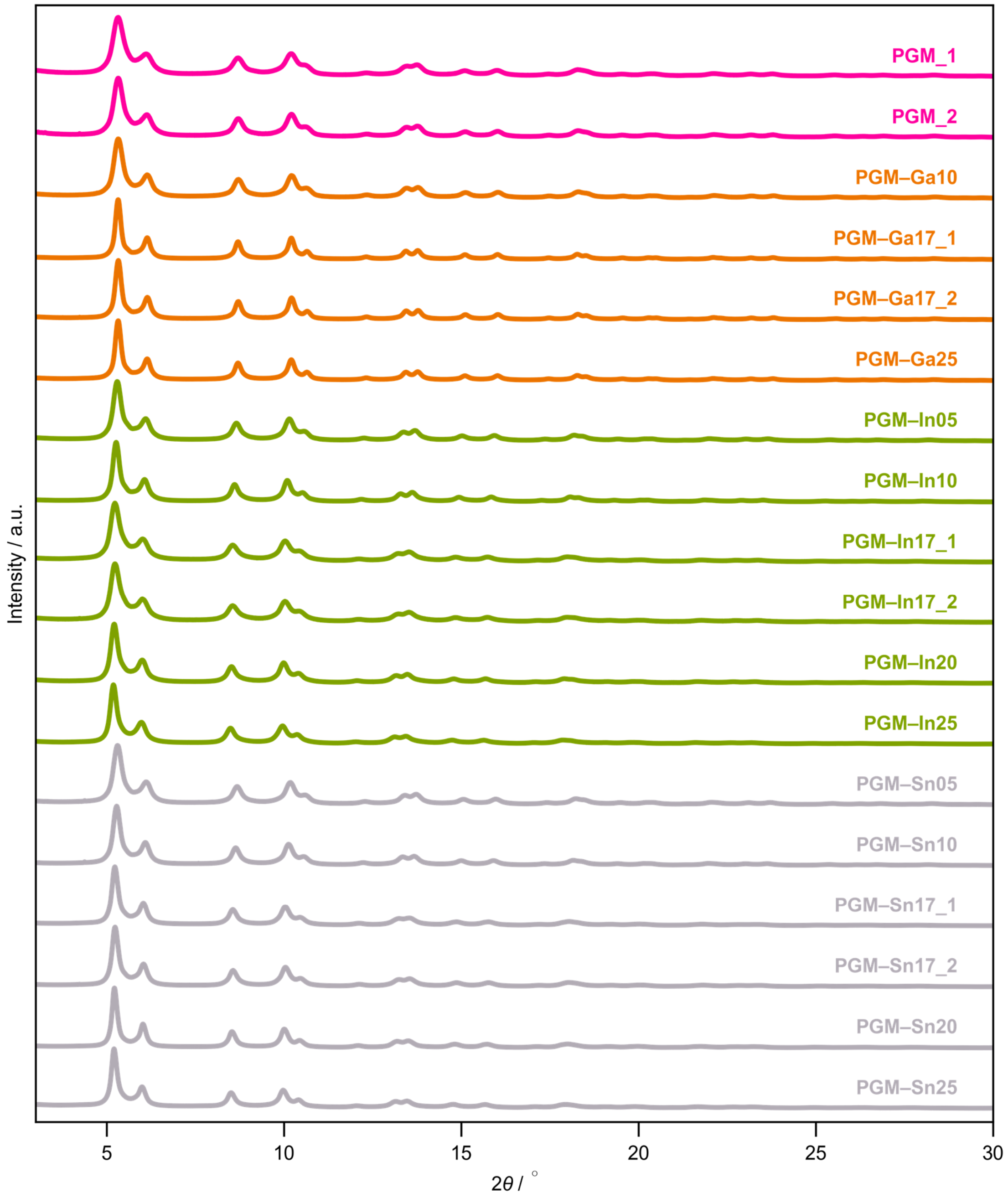

Figure S6 XRD profiles of different samples acquired at 105 K. The incident X-ray energy was 60 keV.

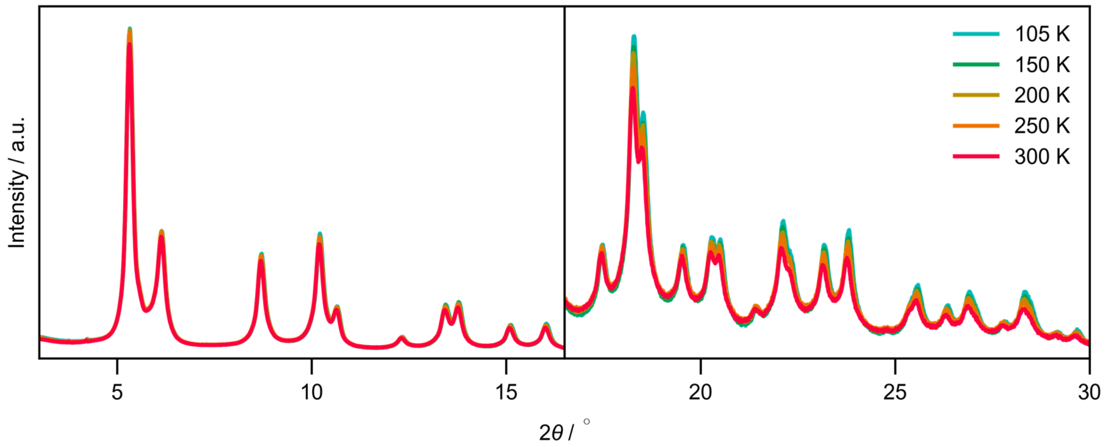

Figure S7 Temperature-dependent XRD profiles of PGM–Ga17_2. Vertical-axis scale is magnified in the high-θ region for clarity.

§2.2. Rietveld Refinement

The XRD profiles were analyzed by Rietveld refinement using the GSAS-II software.[4] The instrumental parameters, including the incident X-ray wavelength, were determined by fitting the profile of the CeO_2 standard. The model structure was an fcc structure occupied by multiple elements, with the occupancies set according to the composition estimated using XRF spectroscopy (**Table S1**). The refined parameters were the lattice parameter *a*, crystallite size (isotropic), microstrain (isotropic), displacement parameter U_{iso} (isotropic), orientation parameter (unique axis along 111), and background coefficients. U_{iso} was assumed to be the same for all elements. **Figure S8** presents the result of the Rietveld refinement for PGM–Ga17_2 at 105 K as an example, while **Table S3** lists the parameters estimated at 300 K.

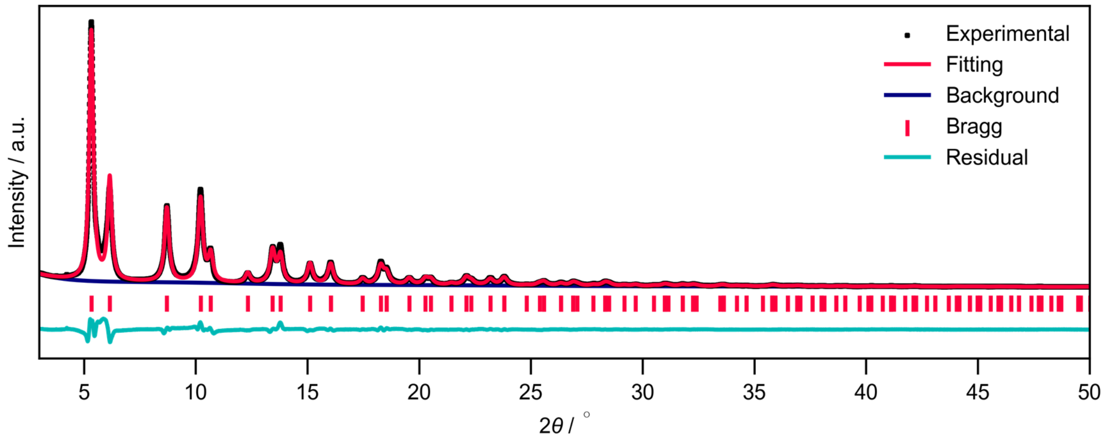

Figure S8 The result of Rietveld refinement obtained for Ga17_2 at 105 K.

Table S3 Structural parameters (lattice parameters *a*, crystallite sizes, and microstrains) estimated at 300 K.

Sample	*a* / Å	Crystallite size / nm	Microstrain / 10^{-2}
**PGM_1**	3.8645(4)	3.95(4)	2.53(7)
**PGM_2**	3.8625(3)	4.09(3)	1.67(6)
**PGM–Ga10**	3.8597(3)	4.59(3)	1.83(5)
**PGM–Ga17_1**	3.8599(2)	8.13(8)	1.95(4)
**PGM–Ga17_2**	3.8592(2)	7.13(6)	1.72(4)
**PGM–Ga25**	3.8608(3)	7.95(8)	2.29(4)
**PGM–In05**	3.8804(3)	5.39(6)	2.64(6)
**PGM–In10**	3.8998(4)	7.2(1)	3.07(6)
**PGM–In17_1**	3.9238(5)	5.66(8)	4.65(9)
**PGM–In17_2**	3.9264(5)	5.73(9)	5.0(1)
**PGM–In20**	3.9386(5)	8.1(2)	4.55(9)
**PGM–In25**	3.9500(5)	12.8(4)	5.90(9)
**PGM–Sn05**	3.8724(3)	4.87(4)	2.13(5)
**PGM–Sn10**	3.8864(3)	6.67(7)	2.98(5)
**PGM–Sn17_1**	3.9219(4)	12.0(3)	4.84(6)
**PGM–Sn17_2**	3.9216(4)	10.3(2)	4.43(6)
**PGM–Sn20**	3.9348(3)	16.4(3)	4.10(4)
**PGM–Sn25**	3.9492(4)	12.0(3)	4.42(8)

§2.3. The Einstein Model and Fitting of Temperature Dependence of MSDs

The Einstein model assumes an ensemble of independent one-dimensional harmonic oscillators with mass m and force constant k. The eigenenergies of a single oscillator can be expressed as

$$E = \left(n + \frac{1}{2}\right)\hbar\sqrt{\frac{k}{m}} \equiv \left(n + \frac{1}{2}\right)\hbar\omega_{\mathrm{E}}$$

where ω_{E} is the eigenfrequency of the oscillator and n is a quantum number. According to the Bose–Einstein statistics, the expected value of the total energy $\langle E\rangle$ per oscillator at finite temperature T is

$$\langle E\rangle = \frac{\hbar\omega_{\mathrm{E}}}{2}\coth\left[\frac{\hbar\omega_{\mathrm{E}}}{2k_{\mathrm{B}}T}\right] = \frac{k_B\theta_E}{2}\coth\left[\frac{\theta_{\mathrm{E}}}{2T}\right] \qquad \textbf{Eq. 1}$$

where θ_{E} is the Einstein temperature defined as

$$\theta_{\mathrm{E}} = \frac{\hbar\omega_{\mathrm{E}}}{k_{\mathrm{B}}} = \frac{\hbar}{k_B}\sqrt{\frac{k}{m}}. \qquad \textbf{Eq. 2}$$

The Einstein temperature θ_{E} is monotonically increases with the force constant k and therefore represents lattice hardness. $\langle E\rangle$ can also be expressed using an MSD. The potential energy term $\langle U\rangle$ can be written using the MSD along a one-dimensional axis $\sigma^2 = \langle x^2\rangle$ as

$$\langle U\rangle = \langle\frac{1}{2}kx^2\rangle = \frac{1}{2}k\sigma^2.$$

Based on the virial theorem, the kinetic term $\langle K\rangle$ is equal to the potential term $\langle U\rangle$ under a harmonic potential. Therefore, the total energy of the system $\langle E\rangle$ should be

$$\langle E\rangle = \langle K\rangle + \langle U\rangle = k\sigma^2. \qquad \textbf{Eq. 3}$$

By combining **Eq. 1** and **Eq. 3**, one can express σ^2 as

$$\sigma^2(T) = \frac{\hbar^2}{2mk_{\mathrm{B}}\theta_{\mathrm{E}}}\coth\left[\frac{\theta_{\mathrm{E}}}{2T}\right].$$

When the static contribution σ^2_{stat} (attributed to lattice strain) is added, the above equation becomes[5]

$$\sigma^2(T) = \frac{\hbar^2}{2mk_{\mathrm{B}}\theta_{\mathrm{E}}}\coth\left[\frac{\theta_{\mathrm{E}}}{2T}\right] + \sigma^2_{\mathrm{stat}}. \qquad \textbf{Eq. 4}$$

The Einstein temperatures of the MEA NPs were estimated by fitting the temperature dependence of U_{iso} obtained via Rietveld refinement, with m was assumed to be the composition-weighted average of the atomic masses. Note that U_{iso} is the MSD projected onto the one-dimensional axis and has the same dimensionality as σ^2 in **Eq. 4**. **Figure S9** shows the results of Einstein model fitting for each sample. **Figure 2c** indicates that the Einstein temperatures decreases as the *p*M molar fraction increases.

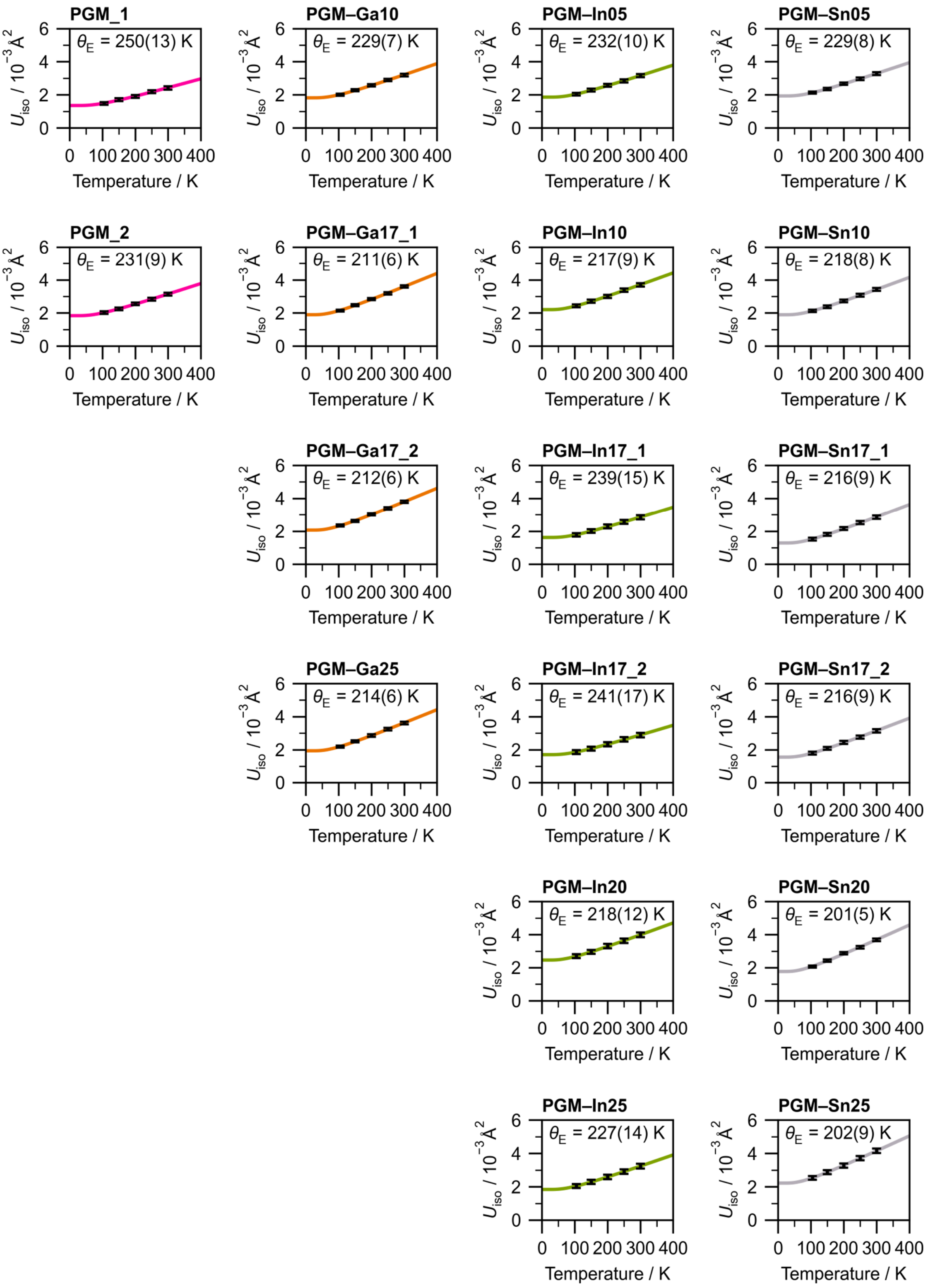

Figure S9 Fitting of the temperature dependence of U_{iso} using the Einstein model. Black error bars represent the values estimated by Rietveld refinement, and the colored lines represent fitting curves.

§2.4. Structural Parameters of Monometals Estimated by XRD

The XRD profiles of the monometals, except for Ga, were acquired and analyzed identically to those of the MEA NPs. The estimated lattice parameters (at 300 K) and Einstein temperatures are summarized in **Table S4**.

Table S4 Estimated structural parameters of the monometals. The atomic radius was calculated as half the first-nearest-neighbor distances at 300 K. The values in parentheses are derived from the second-nearest-neighbor distances when they are close to the first-nearest-neighbor distances.

	Space group	Lattice parameters	Atomic radii / Å	Einstein temp. / K
**Ru**	$P6_3/mmc$	a = 2.70604(2) Å<br>c = 4.28305(3) Å	1.325<br>(1.353)	260(4)
**Rh**	$Fm\overline{3}m$	a = 3.803727(9) Å	1.345	227(1)
**Pd**	$Fm\overline{3}m$	a = 3.89047(2) Å	1.375	151(1)
**Ir**	$Fm\overline{3}m$	a = 3.84036(1) Å	1.358	196(3)
**Pt**	$Fm\overline{3}m$	a = 3.92027(6) Å	1.386	149(1)
**In**	$I4/mmm$	a = 3.25201(6) Å<br>c = 4.9469(2) Å	1.626<br>(1.689)	114(1)
**Sn**	$I4_1/amd$	a = 5.83118(2) Å<br>c = 3.18103(2) Å	1.511<br>(1.591)	91.5(5)

§2.5. Linear Regression of Lattice Parameters with Compositions

To interpret the contribution of the constituent elements to the alloy structures, we assumed that the lattice parameters of the MEA NPs (a_{alloy}) could be expressed by a linear combination of monometallic lattice parameters weighted by composition:

$$a_{\text{alloy}} = \sum_{\text{elem}} x_{\text{elem}} a_{\text{elem}} \qquad \textbf{Eq. 5}$$

where "elem" represents each element in the alloy, x_{elem} is its molar fraction, and a_{elem} is the lattice parameter of the fcc monometals. Assuming a hard-sphere model, a_{elem} can be associated with the atomic radius r_{elem} as

$$a_{\text{elem}} = 2\sqrt{2} r_{\text{elem}}.$$

Thus, **Eq. 5** can be rewritten as

$$a_{\text{alloy}} = 2\sqrt{2} \sum_{\text{elem}} x_{\text{elem}} r_{\text{elem}}. \qquad \textbf{Eq. 6}$$

Although **Eq. 6** can be used to estimate the atomic radius r_{elem} from the experimentally determined a_{alloy} (XRD) and x_{elem} (XRF spectroscopy), the precise determination of r_{elem} for individual PGMs was problematic, as the MEA NPs samples had almost no variation in the PGM ratios. Therefore, the following substitution was made by defining the average atomic radius of PGMs r_{PGM}:

$$\sum_{\text{elem} \in \text{PGM}} x_{\text{elem}} r_{\text{elem}} \rightarrow \left(\sum_{\text{elem} \in \text{PGM}} x_{\text{elem}} \right) r_{\text{PGM}}.$$

Finally, the following equation was used to estimate the r_{elem} of *p*Ms:

$$a_{\text{alloy}} = \left(\sum_{\text{elem} \in \text{PGM}} x_{\text{elem}} \right) a_{\text{PGM}} + \sum_{\text{elem} \in p\text{M}} x_{\text{elem}} a_{\text{elem}}. \qquad \textbf{Eq. 7}$$

Here, x_{elem}s are the values estimated by XRF spectroscopy (**Table S1**), and a_{alloy}s are the values at 300 K (**Table S3**). r_{PGM} was calculated as 1.363 Å by taking the average of two PGM samples (PGM_1 and PGM_2) and fixed during fitting. As reported previously,[6] this value is mostly equal to the average of the atomic radii of the PGM monometals estimated by XRD at 300 K (1.356 Å; **Table S4**). The minimal deviation can be attributed to the interstitial carbon atoms often incorporated in alloy NPs synthesized in an organic solvents at high temperatures.

Figure S10 shows the experimental and predicted values of a_{alloy}, revealing a strong linear correlation between them. **Table S5** lists a_{elem}s and the corresponding r_{elem}s.

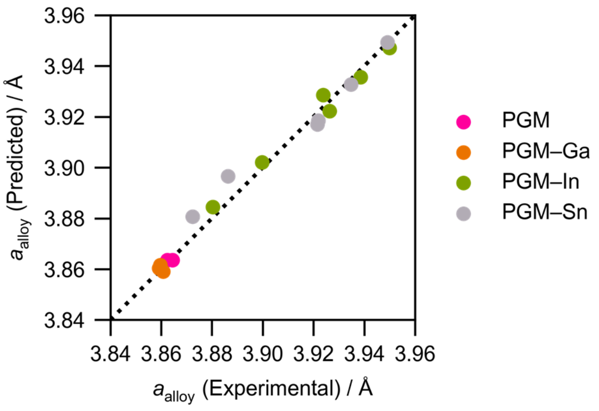

Figure S10 Experimental values of a_{alloy} and those predicted by the linear regression based on **Eq. 7**.

Table S5 Estimated a_{elem}s and corresponding r_{elem}s of *p*Ms.

	a_{elem} / Å	r_{elem} / Å
**Ga**	3.83(2)	1.354(6)
**In**	4.30(2)	1.519(7)
**Sn**	4.17(1)	1.475(4)

§3. EXAFS Analysis of MEA NPs

§3.1. Constraint Formulation

Considering the first-nearest neighbors in the MEA NPs, the scattering paths can be identified by combining the absorption element Abs and the scattering element Scat. Four parameters are considered for each path: the coordination number N, correction term of the absorption energy ΔE_0, bond length R, and MSRD σ^2. Each parameter assigned to the path between Abs and Scat at temperature T is written as

$$\begin{cases} N(\mathrm{Abs}, \mathrm{Scat}, T) \\ \Delta E_0(\mathrm{Abs}, \mathrm{Scat}, T) \\ R(\mathrm{Abs}, \mathrm{Scat}, T) \\ \sigma^2(\mathrm{Abs}, \mathrm{Scat}, T) \end{cases}.$$

Therefore, the original number of parameters is equal to $4n^2t$ for the EXAFS datasets of n-element MEA NPs at t different temperatures.

Several constraints are imposed to reduce the number of parameters.

1) Constraints on N

First, N is considered to be temperature-independent:

$$N(\mathrm{Abs}, \mathrm{Scat}, T) = N(\mathrm{Abs}, \mathrm{Scat}). \quad \textbf{Eq. 8}$$

This is a reasonable assumption, as no structural change is expected at measurement temperatures (up to 373 K) notably lower than those used for the synthesis of the MEA NPs (approximately 573 K). Also, the consistency of the coordination numbers of the equivalent path is preserved by setting

$$x(\mathrm{Abs})N(\mathrm{Abs}, \mathrm{Scat}) = x(\mathrm{Scat})N(\mathrm{Scat}, \mathrm{Abs}) = xN(\mathrm{Scat}, \mathrm{Abs}), \quad \textbf{Eq. 9}$$

where $x(\mathrm{Element})$ represents the composition of each element in the alloy.

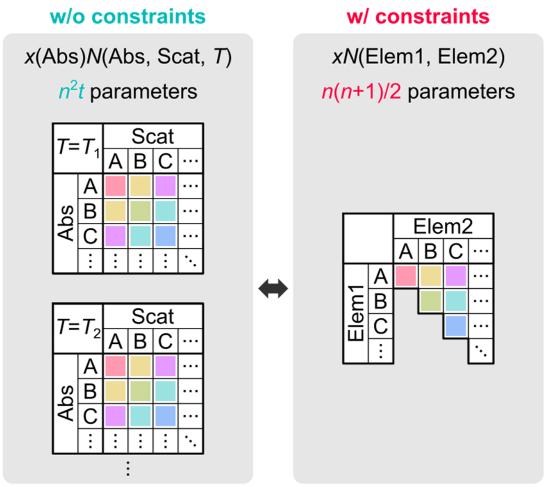

Figure S11 Schematics illustrating the constraints imposed on N. Tables in left and right boxes list the independent fitting parameters without and with the constraints, respectively; each cell represents a parameter in a one-to-one correspondence. The numbers of the parameters are given above the tables, assuming EXAFS datasets of n-element MEA NPs acquired at t temperatures. The parameters shown in the same color are set to have the same values.

2) Constraints on ΔE_0

Second, ΔE_0 is considered to depend only on the absorption element:

$$\Delta E_0(\mathrm{Abs}, \mathrm{Scat}, T) = \Delta E_0(\mathrm{Abs}), \qquad \textbf{Eq. 10}$$

as the chemical shifts among the atoms in the same alloy should not be significantly large. In fact, the XANES analysis (**Figure S5**) shows that the variations of chemical shifts among the samples are in the order of 0.1 eV. In addition, the temperature dependence of the absorption energy can be ignored because quantum statistics predicts that chemical potentials in metallic systems are almost temperature-independent far below the Fermi temperatures, which are typically 10^4 K.

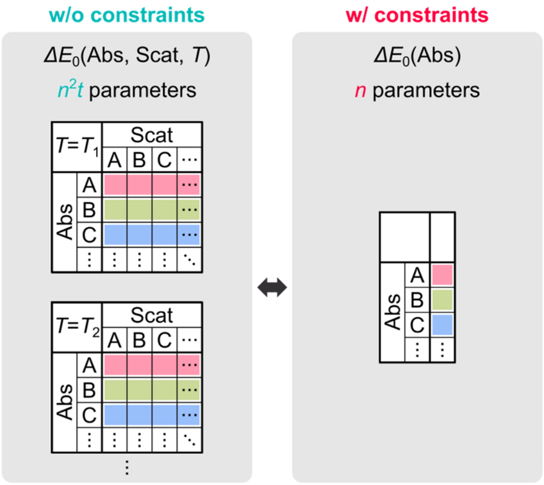

Figure S12 Schematics illustrating the constraints imposed on ΔE_0. Tables in left and right boxes list the independent fitting parameters without and with the constraints, respectively; each cell represents a parameter in a one-to-one correspondence. The numbers of the parameters are given above the tables, assuming EXAFS datasets of *n*-element MEA NPs acquired at *t* temperatures. The parameters shown in the same color are set to have the same values.

3) Constraints on R and σ^2

Third, R is expressed as the sum of the contributions from Abs and Scat:

$$R(\text{Abs}, \text{Scat}, T) = r(\text{Abs}, T) + r(\text{Scat}, T). \quad \textbf{Eq. 11}$$

This can be described within a hard-sphere model, where r is the atomic radius of each element.

Finally, σ^2 is approximated as the sum of the contributions from Abs and Scat:

$$\sigma^2(\text{Abs}, \text{Scat}, T) = \sigma^2_{\text{MSD}}(\text{Abs}, T) + \sigma^2_{\text{MSD}}(\text{Scat}, T). \quad \textbf{Eq. 12}$$

This assumption holds when the two neighboring atoms are independent harmonic oscillators. Although correlation motion cannot be ignored in a real system, this assumption was employed to interpret the individual contributions of each element. Practically, the correlation terms can be considered to be incorporated into the MSDs. The validity of this assumption is discussed in Supporting Information Section **3.9**.

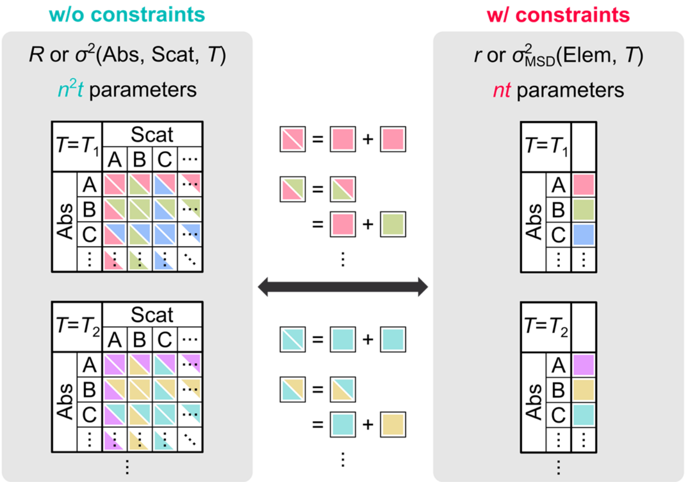

Figure S13 Schematics illustrating the constraints imposed on R and σ^2. Tables in left and right boxes list the independent fitting parameters without and with the constraints, respectively; each cell represents a parameter in a one-to-one correspondence. The numbers of the parameters are given above the tables, assuming EXAFS datasets of n-element MEA NPs acquired at t temperatures. The parameters in the left box, shown in two colors, are assumed to be expressed as the sum of the two parameters shown in the corresponding colors in the right box.

§3.2. Number of Parameters without and with the Constraints

The upper limit of the number of independent parameters in EXAFS fitting is limited by the information content of the experimental data, which is formulated as the Nyquist theorem:

$$N = \frac{2\Delta k \Delta R}{\pi} + m, \qquad \textbf{Eq. 13}$$

where Δk and ΔR are the range of EXAFS data used for fitting in *k*- and *R*-spaces, respectively,[7] and m = 0, 1, or 2 depending on the literature and software. Following the implementation in Larch, $m = 1$ was adopted herein.[8]

To evaluate how the constraint equations (**Eq. 8–12**) can effectively reduce the number of parameters, the number of independent parameters without and with the constraints were compared with the limit defined by **Eq. 13**. For EXAFS data obtained at n absorption edges and t temperatures, the upper limit is

$$N_{\mathrm{lim}} = \left(\frac{2\Delta k \Delta R}{\pi} + 1\right) \times nt. \qquad \textbf{Eq. 14}$$

As mentioned in Section **3.1**, the number of path parameters without constraints is

$$N_{\mathrm{wo}} = 4n^2 t. \qquad \textbf{Eq. 15}$$

In contrast, the number of the independent parameters with the constraints is expressed as

$$N_{\mathrm{w}} = \frac{n(n+1)}{2} + n + 2nt = \frac{n}{2}(n + 4t + 3). \qquad \textbf{Eq. 16}$$

The values of **Eq. 14–16** normalized by the number of data nt are plotted in **Figure 3d**. $\Delta k = 10$ Å^{-1} and $\Delta R = 1.2$ Å were assumed as typical data ranges. For N_{w}, the two cases of $t = 1$ and $t = 7$ were compared. As $N_{\mathrm{wo}} \propto n^2 t$ while $N_{\mathrm{lim}} \propto nt$, N_{wo} inevitably exceeds N_{lim} in MEAs with large n. This problem can be avoided by imposing constraints, as shown in **Figure 3**. The number of parameters can be effectively reduced as t increases, as N_{w} has the order of n^2 and nt while $N_{\mathrm{wo}} \propto n^2 t$.

§3.3. Details of EXAFS Fitting Procedure

EXAFS fitting was performed using an originally developed Python script using the xraylarch (Larch) library[8] version 0.9.65. The following steps were performed before the fitting. First, the S_0^2 parameter (EXAFS amplitude coefficient) of each absorption edge was preliminarily determined by fitting standard samples assuming the chemical transferability (**Table S6**). Second, sample compositions were confirmed based on edge jumps (**Table S7**)[9] and used to impose constraints on coordination numbers (**Eq. 9**). Third, the scattering factors, phase shifts, and mean free paths of the first-nearest-neighbor pairs in the alloy were estimated using FEFF6L[10] implemented in Larch. To calculate the scattering path between Abs and Scat, a cluster modeling the local coordination around Abs in an $L1_2$ alloy ($Scat_3Abs$) up to the fourth coordination shell were used. The interatomic distances were set as the sum of the atomic radii of Abs and Scat, values in **Table S4** for PGMs, and values in **Table S5** for *p*Ms. Finally, the spectra acquired in the QXAFS mode were smoothed with a boxcar filter using the Athena software,[3] following the interpolation of $\chi(k)$ automatically performed in the EXAFS analyses of Athena.

Fitting was performed using the following procedure. First, the spectral data were loaded from Athena project files. Then, EXAFS oscillations were extracted from the XAS spectra using the Autobk algorithm[11] implemented in Larch. The absorption energy E_0 was taken as the energy where the normalized μt equals 0.5 in the lowest-temperature spectrum, and the same value was applied to all spectra acquired at the other temperatures at the same absorption edges. The EXAFS oscillations were Fourier-transformed twice while applying appropriate window functions to extract the contribution of the first-nearest-neighbor pairs in the alloy phase. The background subtraction, forward Fourier transform, and reverse Fourier transform conditions are listed in **Table S8**. Subsequently, each EXAFS spectrum was associated with the appropriate scattering paths calculated using FEFF. In addition, the constraints among the parameters N, ΔE_0, R, and, σ^2 describing each path were imposed following **Eq. 8–12**. To obtain reasonable results, the lower bounds of the coordination numbers were set to zero. Finally, EXAFS fitting was performed in *q*-space using two cycles, not least because the presence of paths with close-to-zero coordination numbers sometimes made error bar estimation difficult. The change in the cost function when each coordination number was set to zero was calculated after the first cycle, and the coordination number was fixed at zero in the next cycle if the relative change was one order of magnitude smaller than the convergence criterion of the fitting.

Table S6 Estimated S_0^2 parameter and the standards used for estimation.

	Standard	S_0^2
**Ru K**	Ru	0.89(4)
**Rh K**	Rh	0.92(4)
**Pd K**	Pd	0.89(4)
**Ir L_3**	Ir	0.83(3)
**Pt L_3**	Pt	0.86(2)
**Ga K**	GaN	0.89(11)
**In K**	In_2O_3	0.85(6)
**Sn K**	SnO_2	0.97(9)

Table S7 Sample compositions (mol%) estimated based on edge jumps.

Sample	Ru	Rh	Pd	Ir	Pt	Ga	In	Sn
**PGM_2**	21.1	20.3	20.7	20.2	17.8	-	-	-
**PGM–Ga17_2**	17.4	19.3	17.7	17.8	15.6	12.2	-	-
**PGM–In17_2**	17.4	18.0	17.3	17.5	15.6	-	14.2	-
**PGM–Sn17_2**	18.0	17.9	16.4	16.1	14.3	-	-	17.4

Table S8 Background subtraction, forward Fourier transform, and reverse Fourier transform conditions. The condition for the same absorption edge was unified even when analyzing different MEA NPs. The index of each row represents a parameter name of the functions in xraylarch.

	Ru K	Rh K	Pd K	Ir L_3	Pt L_3	Ga K	In K	Sn K
**Background subtraction (larch.xafs.autobk)**								
**rbkg**	1.0	1.0	1.0	1.0	1.0	1.0	1.0	1.0
**kmin**	0.0	0.0	0.0	0.0	0.0	0.0	0.0	0.0
**kmax**	16.5	16.5	16.5	8.8	16.5	14.0	16.5	16.5
**Forward Fourier transform (larch.xafs.xftf and larch.xafs.feffit_transform)**								
**kweight**	3	3	3	3	3	3	3	3
**kmin**	4.0	4.0	4.0	3.0	4.0	3.0	4.0	4.0
**kmax**	15.0	15.0	15.0	8.3	15.0	11.0	15.0	15.0
**dk**	1.0	1.0	1.0	1.0	1.0	1.0	1.0	1.0
**window**	hanning	hanning	hanning	hanning	hanning	hanning	hanning	hanning
**Reverse Fourier transform (larch.xafs.xftr and larch.xafs.feffit_transform)**								
**rmin**	1.8	1.8	1.8	1.5	2.1	1.8	2.0	2.0
**rmax**	3.0	3.1	3.1	3.4	3.2	3.3	3.2	3.2
**dr**	0.1	0.1	0.1	0.1	0.1	0.1	0.1	0.1
**window**	hanning	hanning	hanning	hanning	hanning	hanning	hanning	hanning

§3.4. Experimental EXAFS Data

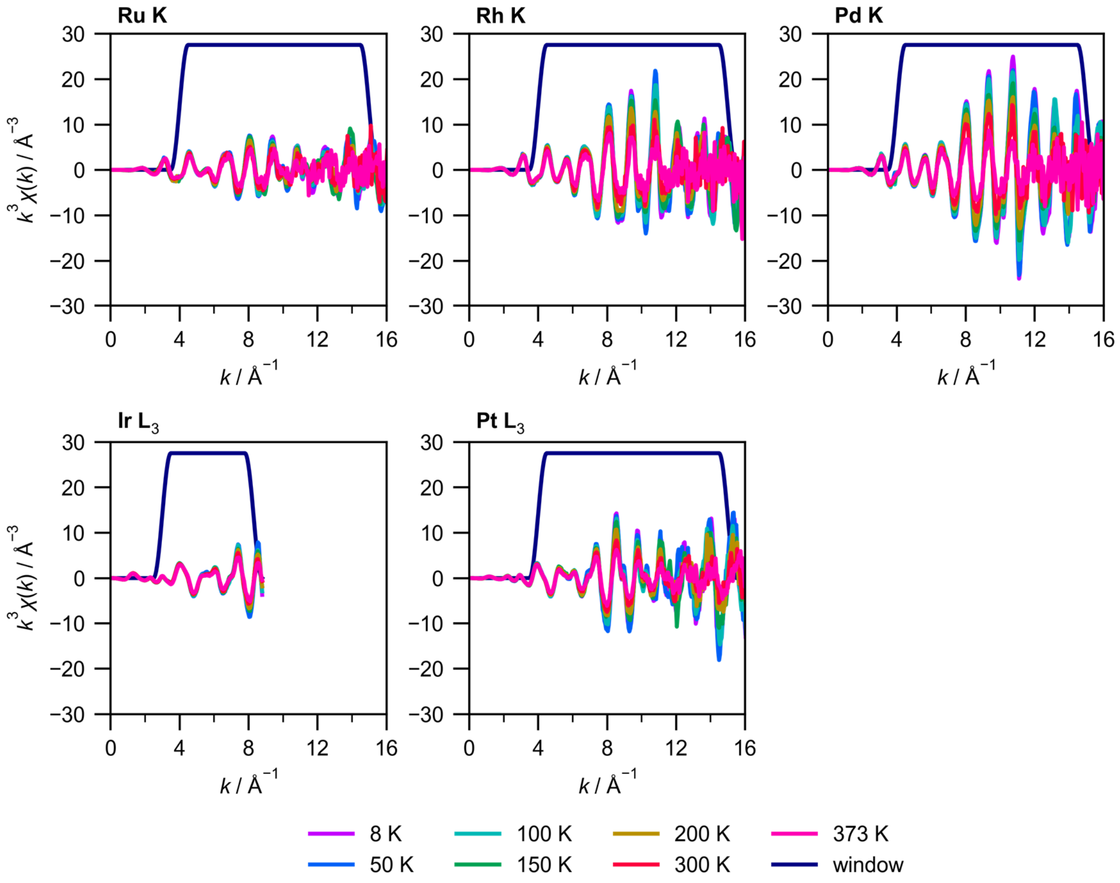

Figure S14 EXAFS oscillations of PGM_2 at all absorption edges and temperatures.

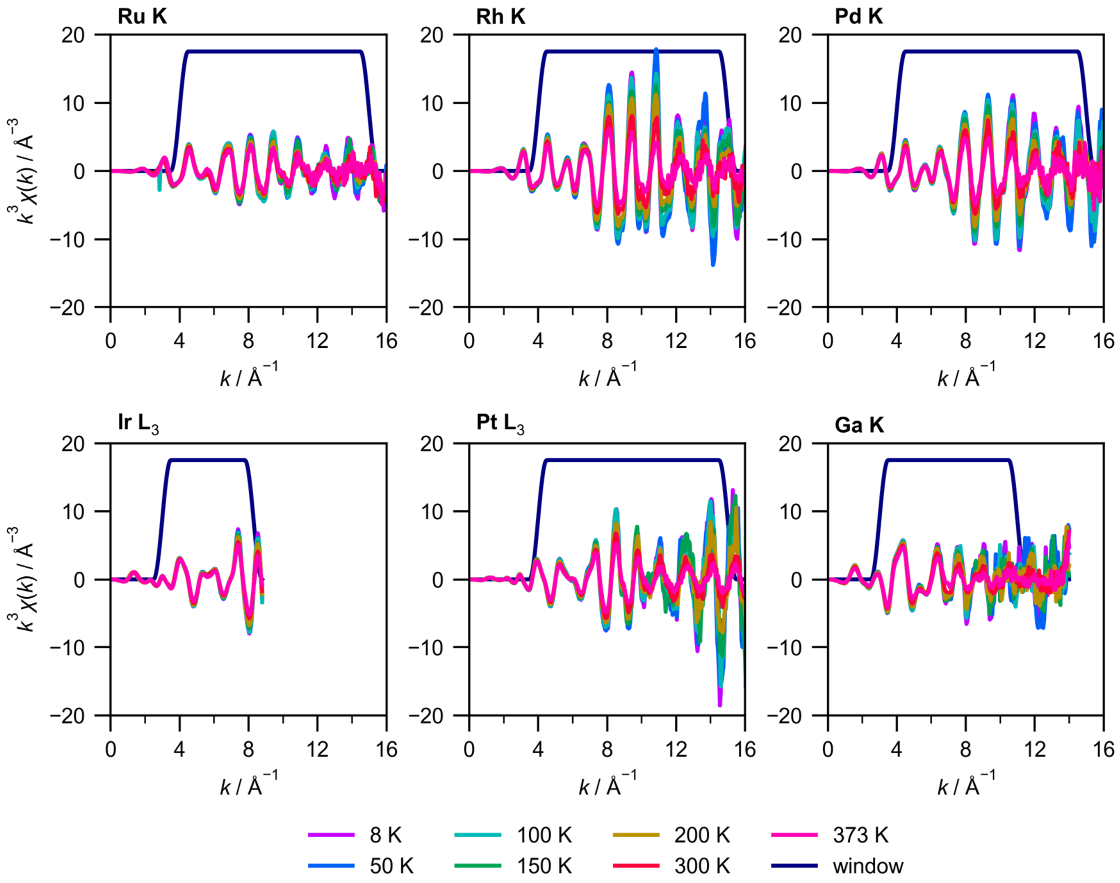

Figure S15 EXAFS oscillations of PGM–Ga17_2 at all absorption edges and temperatures.

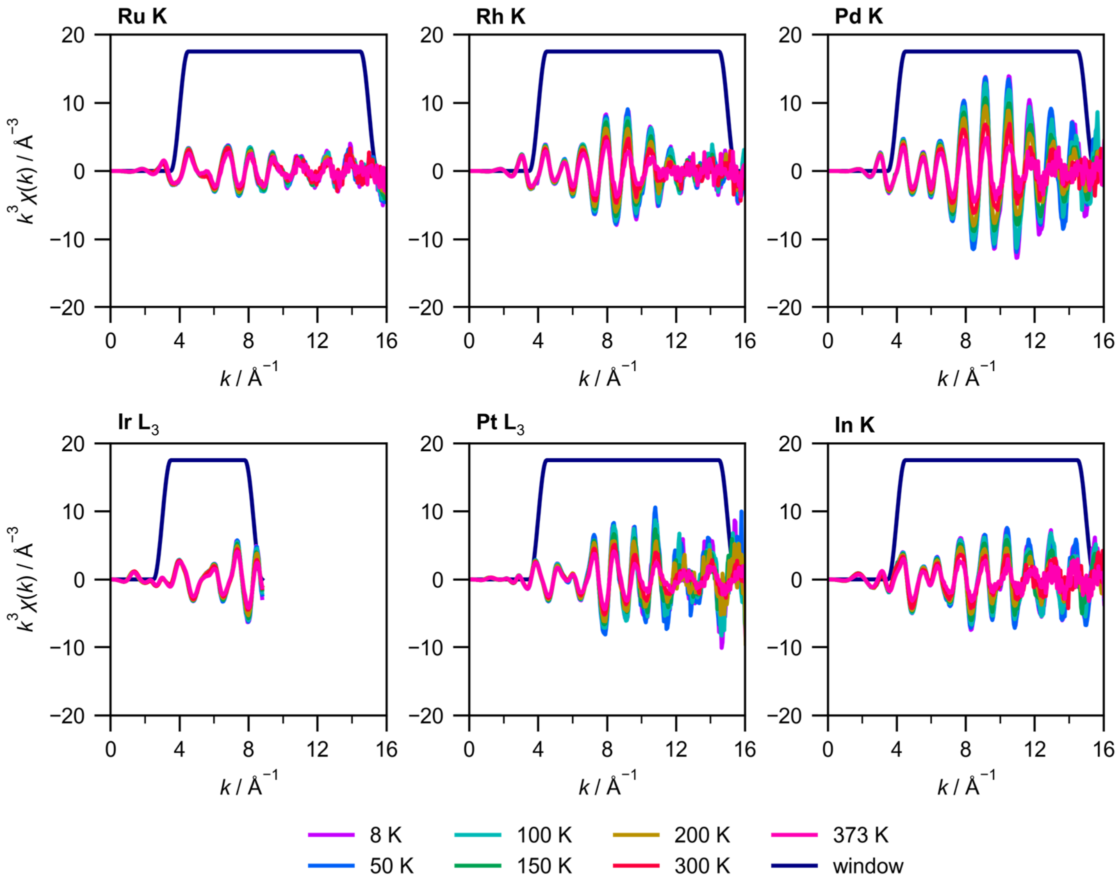

Figure S16 EXAFS oscillations of PGM–In17_2 at all absorption edges and temperatures.

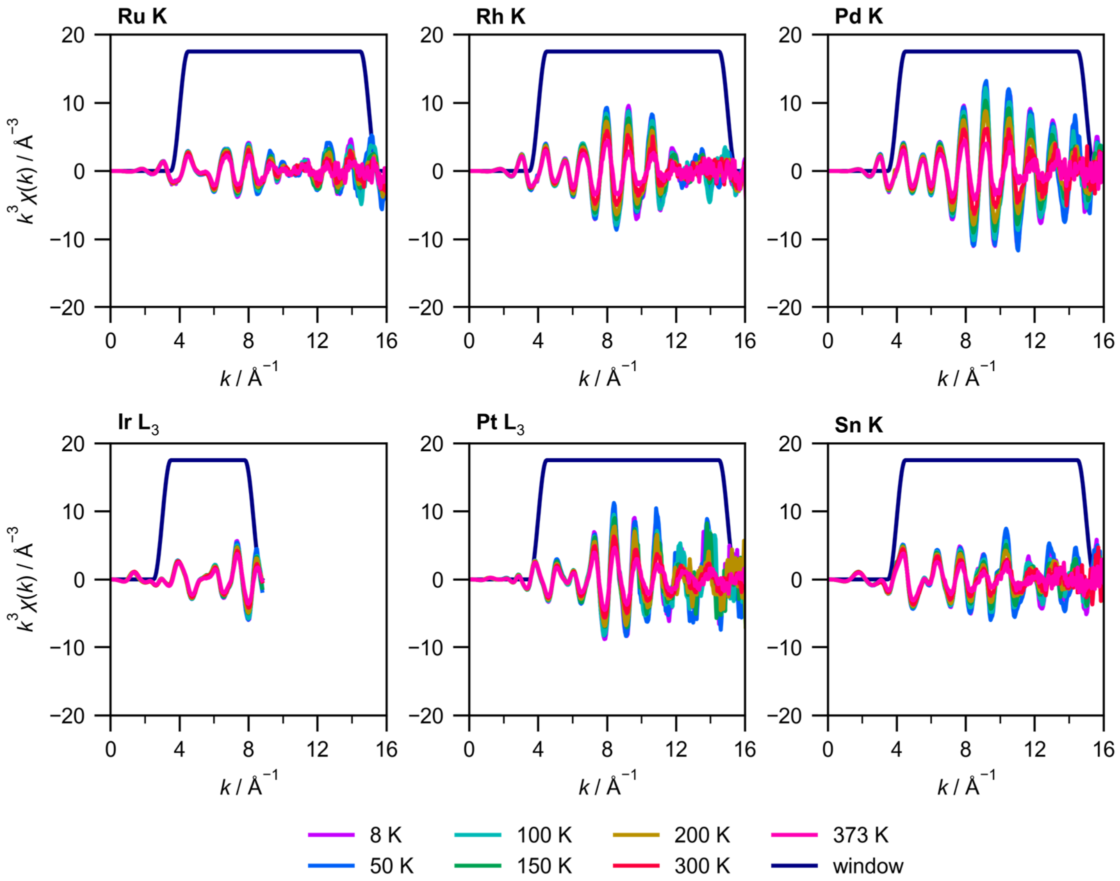

Figure S17 EXAFS oscillations of PGM–Sn17_2 at all absorption edges and temperatures.

§3.5. EXAFS Fitting Results

Table S9, **Figures S18–20** (PGM_2, PGM–Ga17_2, and PGM–In17_2), and **Figure 4** (PGM–Sn17_2) show the results of EXAFS fitting. The fitting was performed using a laptop. The *R* factor is a fitting quality indicator defined as

$$R = \frac{|\boldsymbol{y}_{\mathrm{data}} - \boldsymbol{y}_{\mathrm{fit}}|^2}{|\boldsymbol{y}_{\mathrm{data}}|^2}, \qquad \textbf{Eq. 17}$$

where $\boldsymbol{y}_{\mathrm{data}}$ refers to the experimental data, and $\boldsymbol{y}_{\mathrm{fit}}$ refers to the fitting curve.

The estimated coordination numbers are summarized in **Tables S10–S13**. The estimated atomic radii at 300 K are shown in **Figure 4a**. The estimated MSDs are shown in **Figures S21–24** and were used to estimate the Einstein temperatures.

Table S9 EXAFS fitting times and statistics. N_{lim} is the limit due to the data information (**Eq. 13**). N_{wo} and N_{w} are the numbers of parameters without (**Eq. 15**) and with (**Eq. 16** subtracted by the number of parameters removed based on the result of the first cycle) the constraints. *R* factors (**Eq. 17**) are fitting quality indicators.

	1st cycle	2nd cycle				
	Time (mm:ss)	Time (mm:ss)	N_{lim}	N_{wo}	N_{w}	*R* factor
**PGM_2**	20:44	00:58	320.1	700	88	0.0053
**PGM–Ga17_2**	46:28	01:51	380.5	1008	109	0.0063
**PGM–In17_2**	76:28	02:01	385.9	1008	108	0.0052
**PGM–Sn17_2**	46:00	01:52	385.9	1008	109	0.0043

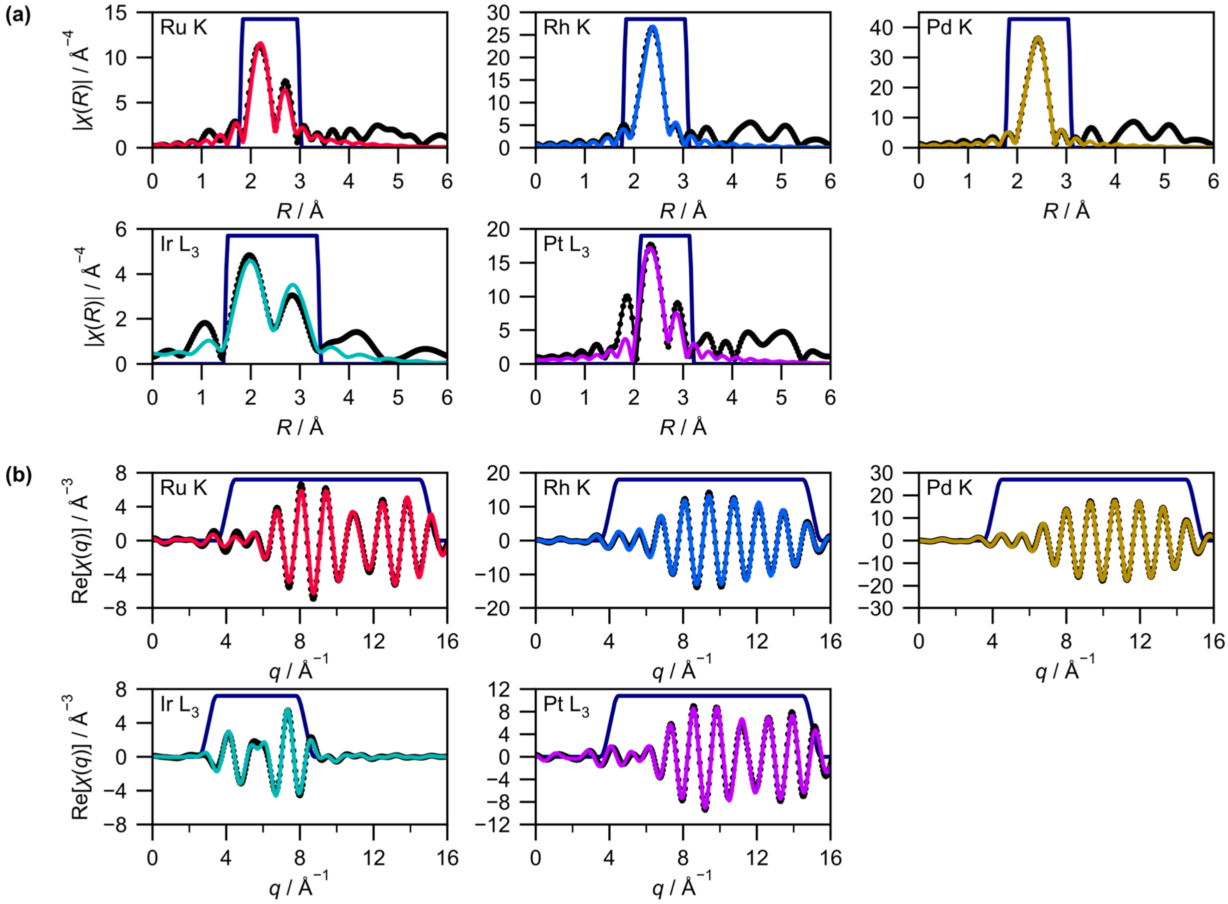

Figure S18 EXAFS fitting results of PGM_2 in **(a)** *R*-space and **(b)** *q*-space. Data acquired at 8 K are selected as examples. Black dots represent the experimental data, while the colored lines represent the fitting curves. Navy lines represent Fourier transform windows.

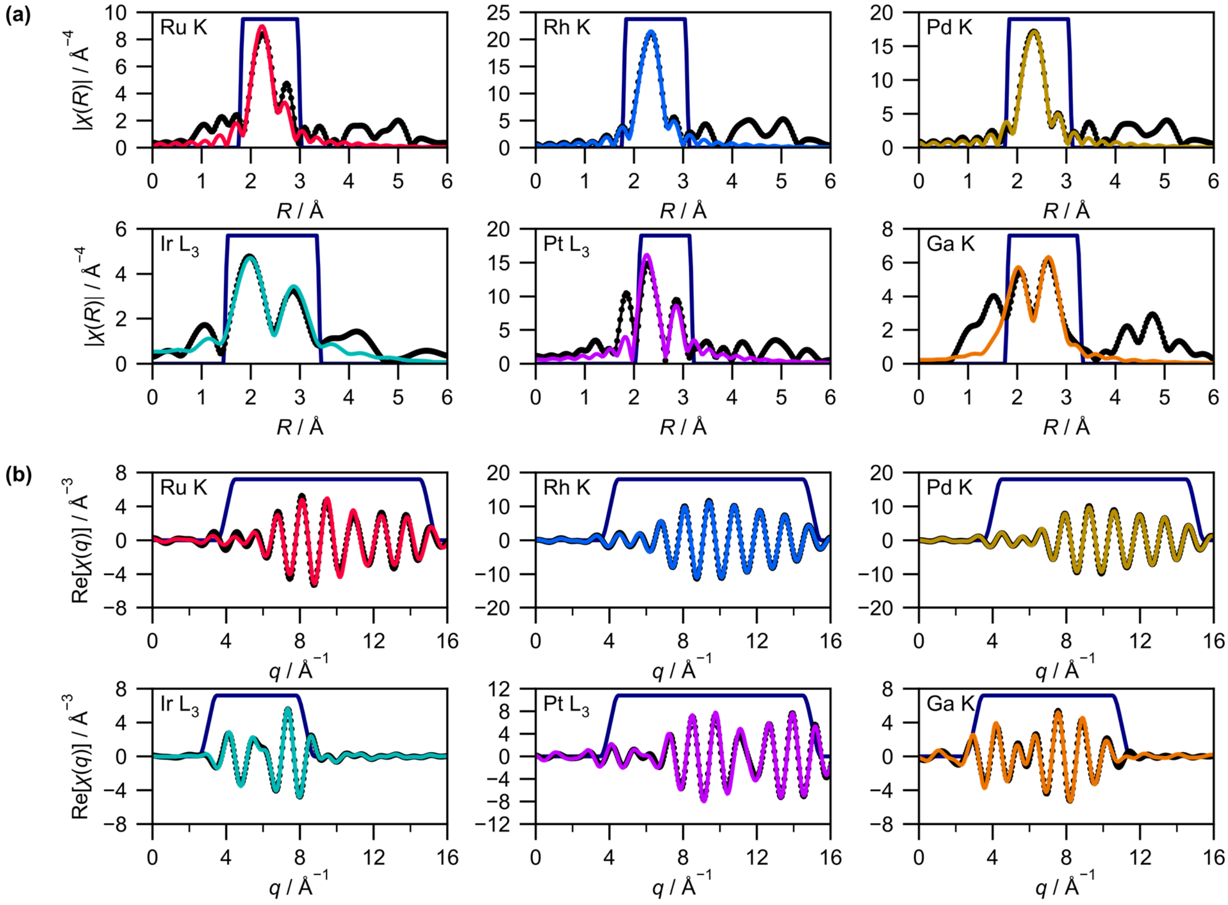

Figure S19 EXAFS fitting results of PGM–Ga17_2 in **(a)** *R*-space and **(b)** *q*-space. Data acquired at 8 K are selected as examples. Black dots represent the experimental data, while the colored lines represent the fitting curves. Navy lines represent Fourier transform windows.

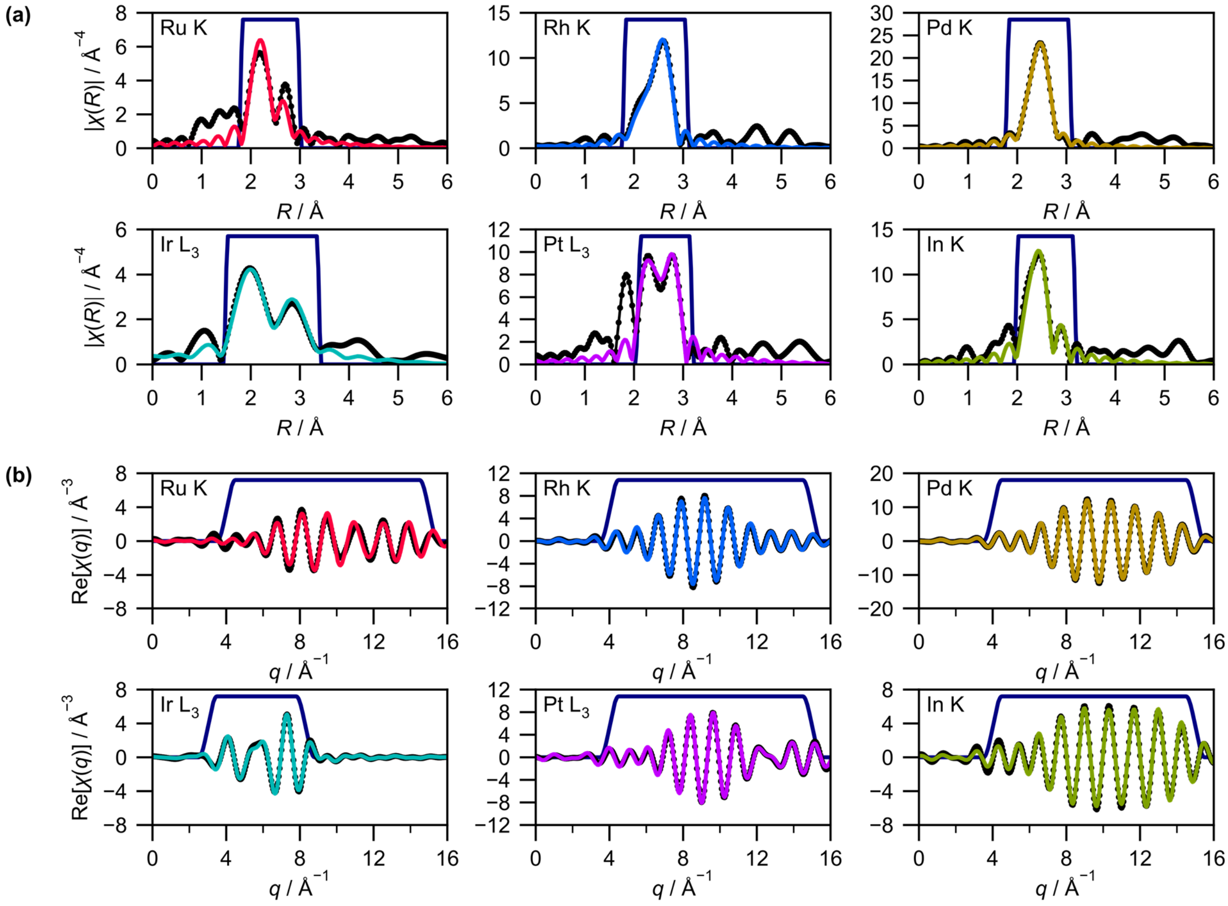

Figure S20 EXAFS fitting results of PGM–In17_2 in **(a)** *R*-space and **(b)** *q*-space. Data acquired at 8 K are selected as examples. Black dots represent the experimental data, while the colored lines represent the fitting curves. Navy lines represent Fourier transform windows.

Table S10 Estimated coordination numbers in PGM_2. The values in parentheses are error bars. The pairs indicated by hyphens were removed from fitting in the second cycle based on the result of the first cycle.

		Scat				
		Ru	Rh	Pd	Ir	Pt
Abs	Ru	0.82 (0.19)	1.92 (0.20)	-	2.90 (0.33)	0.30 (0.19)
	Rh	2.00 (0.21)	3.18 (1.46)	3.67 (1.22)	-	2.37 (0.28)
	Pd	-	3.60 (1.20)	5.05 (1.20)	0.36 (0.28)	1.51 (0.23)
	Ir	3.03 (0.35)	-	0.37 (0.29)	7.33 (1.11)	0.00 (1.06)
	Pt	0.36 (0.23)	2.70 (0.32)	1.75 (0.27)	0.00 (1.20)	2.40 (0.87)

Table S11 Estimated coordination numbers in PGM–Ga17_2. The values in parentheses are error bars. The pairs indicated by hyphens were removed from fitting in the second cycle based on the result of the first cycle.

		Scat					
		Ru	Rh	Pd	Ir	Pt	Ga
Abs	Ru	0.04 (0.26)	2.22 (0.22)	-	1.15 (0.11)	1.00 (0.16)	-
	Rh	1.99 (0.19)	2.23 (0.36)	1.59 (0.41)	1.72 (0.13)	0.83 (0.08)	0.86 (0.22)
	Pd	-	1.74 (0.44)	4.34 (0.57)	0.73 (0.12)	1.66 (0.13)	2.67 (0.27)
	Ir	1.12 (0.11)	1.86 (0.14)	0.73 (0.12)	2.57 (0.23)	1.21 (0.08)	2.65 (0.24)
	Pt	1.12 (0.18)	1.03 (0.09)	1.89 (0.14)	1.38 (0.09)	1.10 (0.15)	2.56 (0.32)
	Ga	-	1.36 (0.34)	3.88 (0.40)	3.88 (0.35)	3.28 (0.41)	1.90 (0.75)

Table S12 Estimated coordination numbers in PGM–In17_2. The values in parentheses are error bars. The pairs indicated by hyphens were removed from fitting in the second cycle based on the result of the first cycle.

		Scat					
		Ru	Rh	Pd	Ir	Pt	In
Abs	Ru	0.81 (0.25)	0.15 (0.32)	0.58 (0.27)	1.37 (0.22)	-	0.21 (0.19)
	Rh	0.15 (0.31)	1.97 (0.75)	0.29 (0.65)	0.96 (0.23)	1.26 (0.15)	3.28 (0.32)
	Pd	0.58 (0.27)	0.30 (0.67)	6.36 (0.66)	-	1.47 (0.11)	0.28 (0.25)
	Ir	1.36 (0.21)	0.99 (0.24)	-	7.50 (0.35)	-	0.00 (0.13)
	Pt	-	1.45 (0.17)	1.64 (0.12)	-	2.02 (0.22)	1.79 (0.20)
	In	0.25 (0.23)	4.15 (0.41)	0.35 (0.31)	0.00 (0.16)	1.97 (0.22)	0.65 (0.31)

Table S13 Estimated coordination numbers in PGM–Sn17_2. The values in parentheses are error bars. The pairs indicated by hyphens were removed from fitting in the second cycle based on the result of the first cycle.

		Scat					
		Ru	Rh	Pd	Ir	Pt	Sn
Abs	Ru	2.07 (0.27)	-	-	2.26 (0.29)	0.38 (0.25)	1.24 (0.17)
	Rh	-	2.67 (0.42)	1.85 (0.36)	0.91 (0.22)	1.62 (0.17)	1.96 (0.22)
	Pd	-	2.02 (0.40)	4.65 (0.51)	0.00 (0.23)	2.10 (0.22)	0.23 (0.16)
	Ir	2.51 (0.32)	1.01 (0.24)	0.00 (0.23)	5.88 (0.69)	0.00 (0.59)	0.00 (0.16)
	Pt	0.47 (0.31)	2.02 (0.21)	2.40 (0.25)	0.00 (0.66)	1.97 (0.43)	2.17 (0.35)
	Sn	1.28 (0.17)	2.01 (0.22)	0.22 (0.15)	0.00 (0.15)	1.79 (0.29)	1.13 (0.30)

§3.6. Fitting of Element-Dependent MSDs to the Einstein model

The temperature dependence of $\sigma^2_{\mathrm{MSD}}(\mathrm{elem}, T)$ estimated by the EXAFS fitting was fitted using the Einstein model (**Eq. 4**) to estimate element-dependent Einstein temperatures.

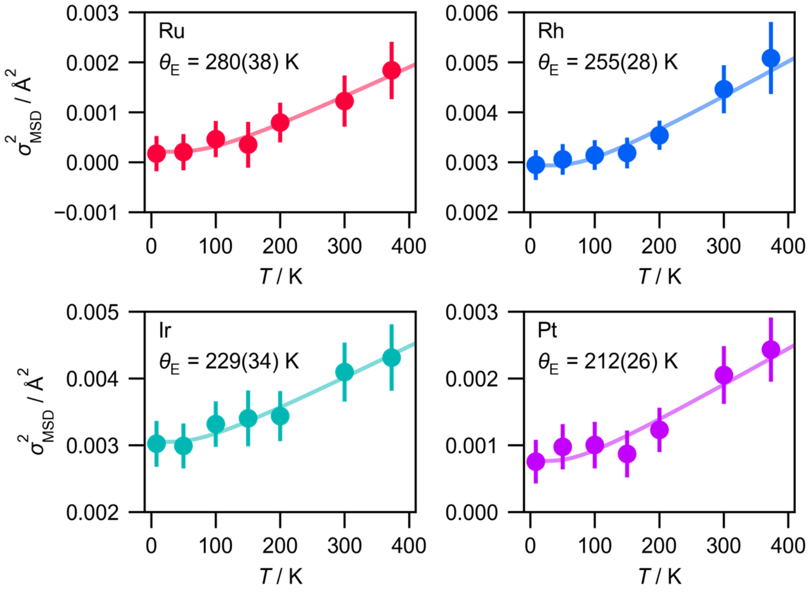

Figure S21 Temperature dependence of $\sigma^2_{\mathrm{MSD}}(\mathrm{elem}, T)$ in PGM_2 estimated by EXAFS analysis (markers with error bars) and fitting to the Einstein model (line). Each graph shows $\sigma^2_{\mathrm{MSD}}(\mathrm{elem}, T)$ assigned to each element.

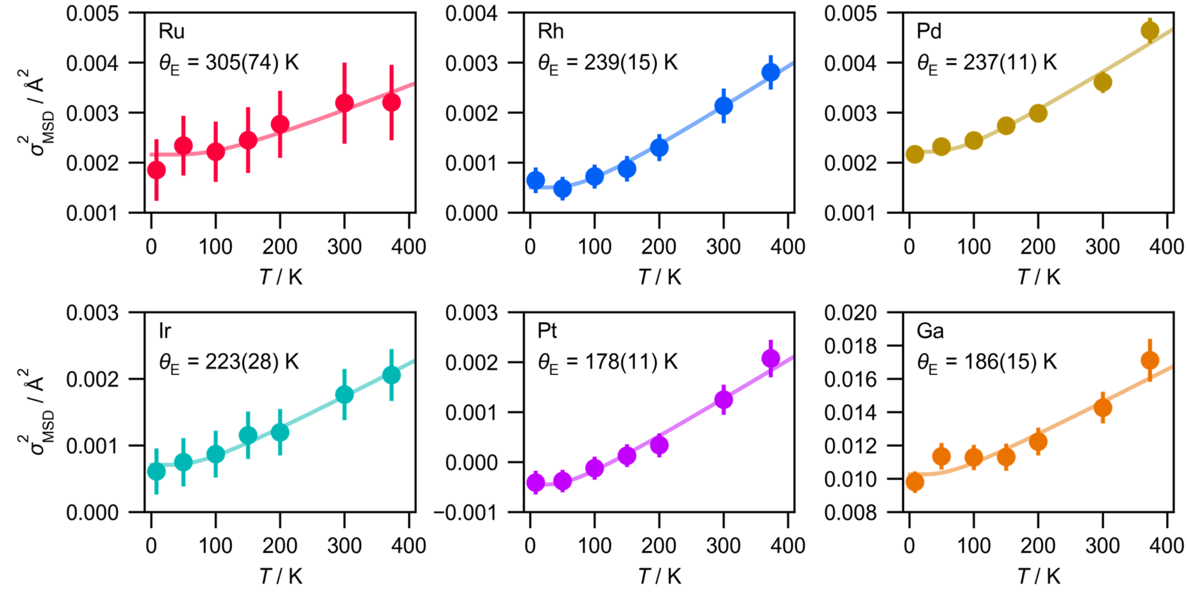

Figure S22 Temperature dependence of $\sigma^2_{\mathrm{MSD}}(\mathrm{elem}, T)$ in PGM–Ga17_2 estimated by EXAFS analysis (markers with error bars) and fitting to the Einstein model (line). Each graph shows $\sigma^2_{\mathrm{MSD}}(\mathrm{elem}, T)$ assigned to each element.

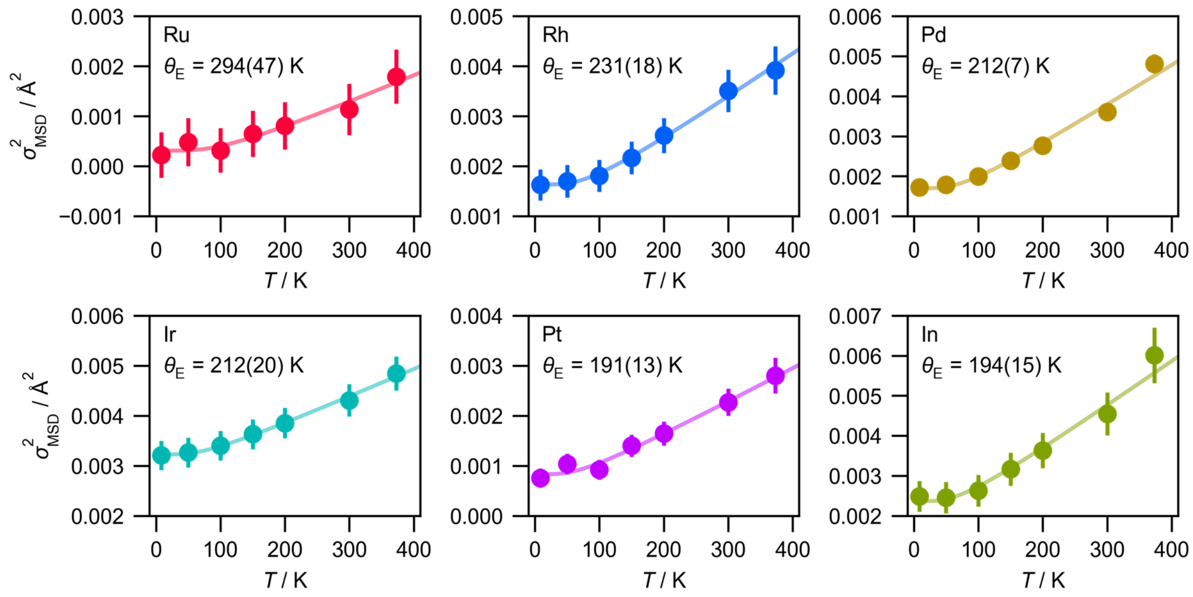

Figure S23 Temperature dependence of $\sigma^2_{\mathrm{MSD}}(\mathrm{elem}, T)$ in PGM–In17_2 estimated by EXAFS analysis (markers with error bars) and fitting to the Einstein model (line). Each graph shows $\sigma^2_{\mathrm{MSD}}(\mathrm{elem}, T)$ assigned to each element.

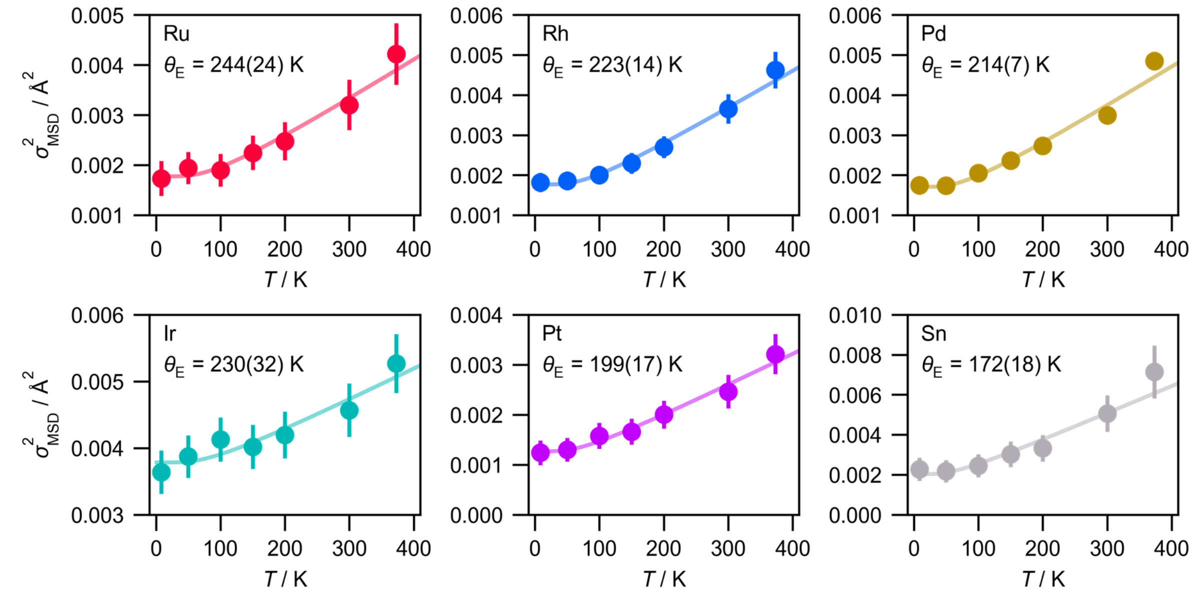

Figure S24 Temperature dependence of $\sigma^2_{\mathrm{MSD}}(\mathrm{elem}, T)$ in PGM–Sn17_2 estimated by EXAFS analysis (markers with error bars) and fitting to the Einstein model (line). Each graph shows $\sigma^2_{\mathrm{MSD}}(\mathrm{elem}, T)$ assigned to each element.

§3.7. Consideration on Correlation between Coordination Number and MSRD

To discuss the effect of the correlation between the coordination number and MSRD on the estimation of the Einstein temperature, we fitted the simulated EXAFS spectra of monometallic Pt. Data were obtained at the seven temperatures used for EXAFS measurements. The coordination number N was set to 12. The Einstein temperature was assumed to be 200 K, and σ^2 (MSRD) was calculated using **Eq. 4** (Einstein model) and **Eq. 12** (MSRD = sum of MSDs). Then, fitting was performed while fixing N at 4–20. The estimated temperature dependence of σ^2 is shown in **Figure S25a**. Finally, the Einstein temperatures θ_E were derived. **Figure S25b** shows the θ_E values estimated for different N. Although systematic errors were found, their scale was approximately 5% of the genuine value (200 K) even when the error of N was ≥50%. Therefore, we concluded that the Einstein temperature can be evaluated within a small error even when the absolute values of σ^2s are wrong because of the correlation with N.

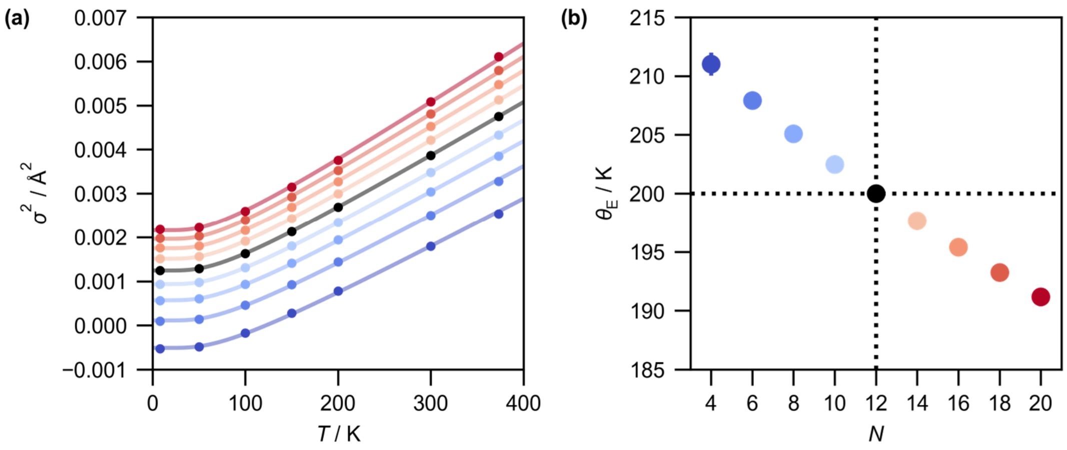

Figure S25 **(a)** Temperature dependence of MSRD σ^2 estimated by fitting simulated EXAFS spectra (N = 12 and θ_E = 200 K) while fixing N at 4–20. Lines represent fits to the Einstein model. Different colors represent fits obtained for different N. **(b)** Einstein temperatures θ_E estimated for different N, with the color code corresponding to that in (a). Dotted lines represent the genuine values of N and θ_E assumed in the initial simulation.

§3.8. EXAFS Analyses of Monometals

The temperature-dependent EXAFS of the monometallic PGMs were acquired at six temperatures (8, 50, 100, 150, 200, and 300 K). The data were analyzed as for the MEA NPs to derive their atomic radii and Einstein temperatures (**Table S14**).

Figure S26 correlates the Einstein temperatures of the monometallic PGMs estimated by XRD with those estimated by EXAFS. Although the latter values exceed the former, they have a linear relationship. The discrepancy can be ascribed to the anisotropy of the interatomic potential and the neglected correlation motion term in the EXAFS analyses, as discussed in Section **3.9**.

Table S14 Estimated structural parameters of the monometallic PGMs. The atomic radii are the values at 300 K.

	Atomic radii / Å	Einstein temperatures / K
**Ru**	1.3397(6)	293(7)
**Rh**	1.3442(9)	264(6)
**Pd**	1.374(1)	217(5)
**Ir**	1.3570(6)	224(6)
**Pt**	1.3831(5)	178(2)

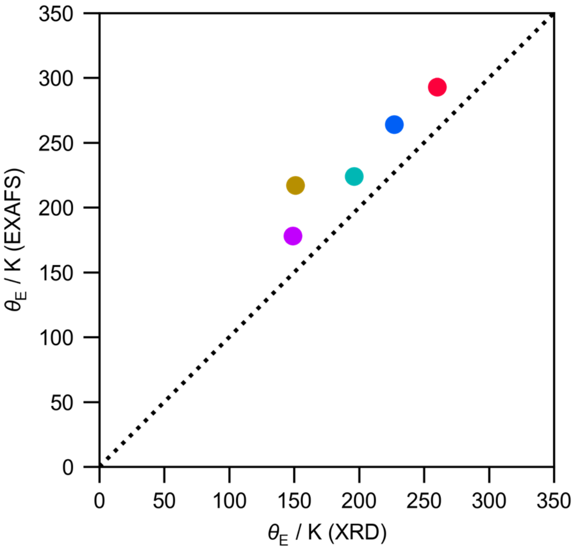

Figure S26 Correlation between the Einstein temperatures of the monometallic PGMs estimated by XRD (**Table S4**) and EXAFS (**Table S14**).

§3.9. Consideration on Correlation Motion Term

The MSRD between atoms X and Y is defined as

$$\sigma^2_{\mathrm{MSRD}}(\mathrm{X},\mathrm{Y}) = \langle (u(\mathrm{X}) - u(\mathrm{Y}))^2 \rangle, \qquad \textbf{Eq. 18}$$

where $u(\mathrm{X})$ and $u(\mathrm{Y})$ are the displacements of the atoms from their equilibrium positions. For simplicity, only atomic motion parallel to the bond axis is considered. **Eq. 18** can be expressed using the MSDs of the atoms $\sigma^2_{\mathrm{MSD}}(\mathrm{X}) = \langle u(\mathrm{X})^2 \rangle$ and $\sigma^2_{\mathrm{MSD}}(\mathrm{Y}) = \langle u(\mathrm{Y})^2 \rangle$ as[5]

$$\sigma^2_{\mathrm{MSRD}}(\mathrm{X},\mathrm{Y}) = \sigma^2_{\mathrm{MSD}}(\mathrm{X}) + \sigma^2_{\mathrm{MSD}}(\mathrm{Y}) - 2\langle u(\mathrm{X})u(\mathrm{Y}) \rangle. \qquad \textbf{Eq. 19}$$

As mentioned previously, the assumption in **Eq. 12** for EXAFS fitting ignores the last term of **Eq. 19**, which represents the correlated motion of the atoms and is often called the displacement correlation function (DCF). In other words, the MSDs estimated by our EXAFS analyses contain the negative contributions from the DCF.

Although we employed the Einstein model, which assumes independent oscillators, to isolate the contributions of different elements, the Debye model is better suited for describing the correlated motion of atoms. According to the Debye model, the MSRD in a monometal can be written as[12]

$$\sigma^2_{\mathrm{MSRD}}(T) = \frac{3\hbar^2}{mk_{\mathrm{B}}\theta_{\mathrm{D}}}\left\{\left(\frac{T}{\theta_{\mathrm{D}}}\right)^2 \Phi_1 + \frac{1}{4}\right\} - \frac{3\hbar^2}{mk_{\mathrm{B}}\theta_{\mathrm{D}}}\left[\frac{1-\cos[q_{\mathrm{D}}R]}{2(q_{\mathrm{D}}R)^2} + \left(\frac{T}{\theta_{\mathrm{D}}}\right)^2\left\{\Phi_1 - \frac{1}{3!}\left(q_{\mathrm{D}}R\frac{T}{\theta_{\mathrm{D}}}\right)^2\Phi_3 + \frac{1}{5!}\left(q_{\mathrm{D}}R\frac{T}{\theta_{\mathrm{D}}}\right)^4\Phi_5 - \cdots\right\}\right], \qquad \textbf{Eq. 20}$$

where θ_{D} is the Debye temperature, q_D is the radius of the Brillouin zone in the Debye model, R is the interatomic distance, and Φ_n is

$$\Phi_n = \int_0^{\theta_{\mathrm{D}}/T} \mathrm{d}x \frac{x^n}{\exp[x]-1}.$$

The first term of **Eq. 20** represents the sum of the MSDs, and the second term represents the DCF. Assuming an fcc monometal ($q_{\mathrm{D}}R \approx 4.375$ for the first-nearest-neighbor pair), the MSD and the DCF were calculated as depicted in **Figure S27**. In this model, the DCF is as large as 15–40% of the MSD.

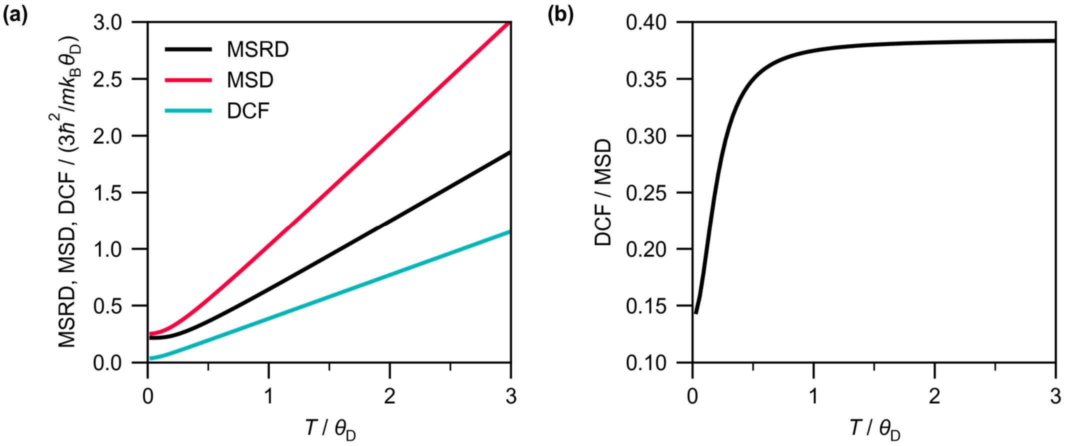

Figure S27 Temperature dependence of **(a)** MSRD, MSD, and DCF of the first-nearest-neighbor pair in an fcc monometal calculated using the Debye model (**Eq. 20**) and **(b)** the DCF/MSD ratio.

The influence of the DCF contribution on the estimation of the Einstein temperatures is discussed below. The MSDs and MSRDs at the seven temperatures used for the EXAFS measurements were calculated using the Debye model (**Eq. 20**). The temperature dependences of the MSDs and MSRDs were fitted to the Einstein model (**Eq. 4**) to derive the Einstein temperatures $\theta_{E,MSD}$ and $\theta_{E,MSRD}$ (**Figure S28**). This procedure was repeated for various Debye temperatures θ_D, and the correlations among θ_D, $\theta_{E,MSD}$, and $\theta_{E,MSRD}$ were examined (**Figure S29**). Notably, proportionality was observed regardless of θ_D. In this virtual experiment, $\theta_{E,MSD}$ represents the XRD results, while $\theta_{E,MSRD}$ represents the EXAFS results (because a MSRD were simply approximated as a sum of MSDs in our EXAFS analysis). The successful fitting of the MSDs (**Figure S28a**) and MSRDs (**Figure S29b**) to the Einstein model and the linear relationships of $\theta_{E,MSD}$ (**Figure S29a**) and $\theta_{E,MSRD}$ (**Figure S29b**) with θ_D support the validity of using the Einstein model instead of the Debye model in the XRD and EXAFS analysis, respectively. Also, **Figure S29c** explains the discrepancy between the XRD and EXAFS-determined Einstein temperatures. Although the assumption in **Eq. 12**, which incorporates the DCF contribution into the MSD, results in an overestimation of the Einstein temperature (as experimentally shown in **Figure S26**) in the EXAFS analysis, the relative values of the estimated Einstein temperatures are still reliable indicators of dynamic structures.

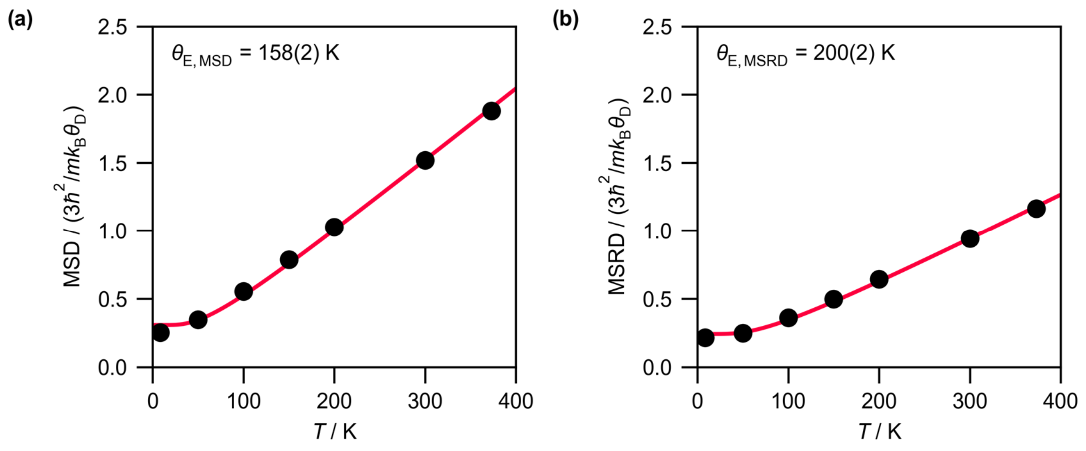

Figure S28 **(a)** MSDs and **(b)** MSRDs of the first-nearest-neighbor pair in an fcc monometal calculated using the Debye model (black markers) at a Debye temperature of 200 K. Red curves represent fits to the Einstein model.

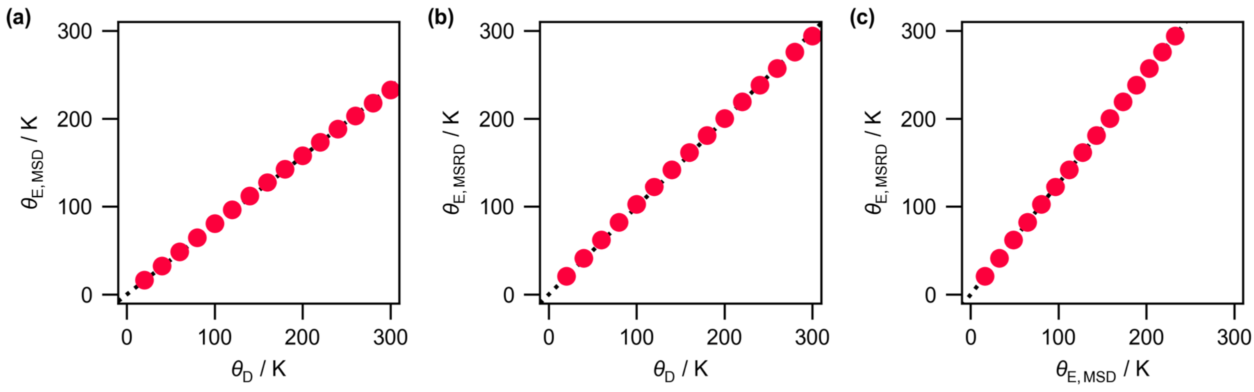

Figure S29 Correlations between **(a)** θ_D and $\theta_{E,MSD}$, **(b)** θ_D and $\theta_{E,MSRD}$, and **(c)** $\theta_{E,MSD}$ and $\theta_{E,MSRD}$, with the corresponding linear fits presented as dotted lines.

§3.10. Consideration on Spectral Overlaps

In MEAs, it would be inevitable that some of the constituent elements have proximate edge energies. For example, Ir L_3 and Pt L_3 edges are only 350 eV apart. The spectral overlaps are problematic for the analysis in two aspects. First, the EXAFS of the element with the lower edge energy (Ir in our case) is interrupted by the subsequent edge. The shortening of k-ranges means a loss of the structural information as represented by **Eq. 13**, which may result in less reliable results. The other problem is that the EXAFS of the element with the higher edge energy (Pt in our case) is contaminated by the preceding edge. Below, the latter effect will be discussed.

To evaluate the influence of the Ir L_3 EXAFS on the Pt L_3 EXAFS, the EXAFS of Ir metal was measured in the energy range of Pt L_3 edge. Then, the spectra of Pt and Ir were summed. The equimolar Ir:Pt ratio was reproduced by normalizing the absorbances (μt) using the edge jumps without pre-edge and post-edge subtraction. The synthesized spectrum was processed and fitted in the perfectly same condition as the MEAs (**Table S8**). **Figure S30** shows the FT-EXAFS of the synthesized spectra and the original Pt spectra. The fitting curves are also shown in the figure, and the corresponding fitting results are summarized in **Table S15**. The most prominent change was the emergence of peaks around 1.5–2.0 Å. All the Pt L_3 FT-EXAFS spectra of the MEA NPs (**Figures 4a** and **S18a–20a**) have the peaks at the same position. Therefore, these artifact peaks were excluded from the fitting range in the EXAFS fitting of the MEA NPs.

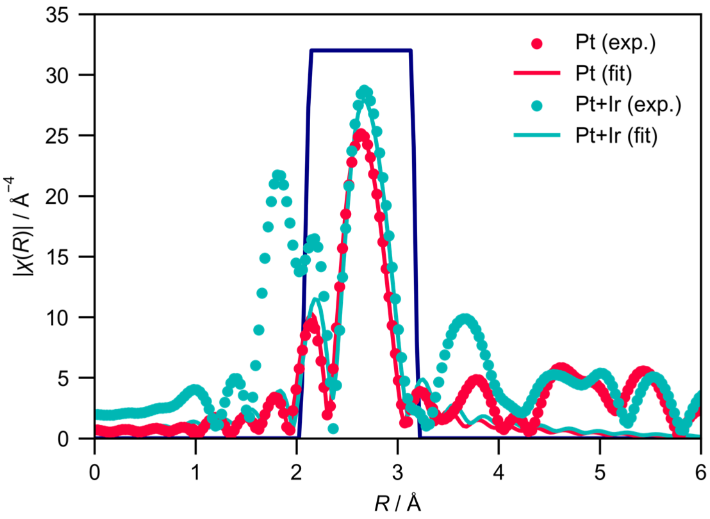

Figure S30 The FT-EXAFS of Pt monometal (Pt) and the synthesized spectra of Pt and Ir (Pt+Ir). The markers represent the experimental data, including the synthesized spectra, and the solid lines represent the fitting curves.

Table S15 The EXAFS fitting results of Pt monometal (Pt) and the synthesized spectra of Pt and Ir (Pt+Ir).

	Pt	Pt+Ir
N	12	11(4)
ΔE_0 / eV	7.0(6)	13(5)
R / Å	2.765(2)	2.79(1)
σ^2 / Å^2	0.0052(2)	0.004(1)

Because the experiment above does not fully reflect the condition of the variable-temperature measurements, the errors arising from the spectral overlap were further estimated using a simulation. The Pt L_3 EXAFS of Pt monometal and the spectra contaminated by Ir L_3 EXAFS of Ir monometal were simulated using FEFF6 implemented in Larch. For simplicity, only the first-nearest neighbors were considered in the simulations. The bond lengths R and MSRDs σ^2 were determined using the atomic radii and the Einstein temperature shown in **Table S14**. The spectra at seven different temperatures used for the EXAFS experiments were simulated. **Figure S31a** shows the simulated FT-EXAFS at 8 K, reproducing the artifact peak. Then, the simulated spectra contaminated by Ir L_3 EXAFS were fitted in the same way as the MEA NPs (**Figure S31b**): the spectra at all the temperatures were simultaneously fitted assuming N and ΔE_0 were temperature independent. Additionally, R was assumed to be temperature independent because the temperature dependence of the atomic radii was not considered in the simulations. **Table S16** shows the parameters (N, ΔE_0, R, and $\sigma^2(T)$) used in the initial simulation and derived from the fitting. The estimated bond length R indicates that the error of the atomic radii estimation (**Figure 5a**) would be 0.02 Å or less. This is small enough to discuss the element dependence of the atomic radii although further improvements are required to discuss the sample dependence of the same element. In contrast, the errors of N and $\sigma^2(T)$ were unignorable. Both N and $\sigma^2(T)$ are underestimated, which is reasonable considering the N–σ^2 correlation. Assumingly, these parameters are susceptible to the spectral overlap because of this correlation. The underestimation is consistent with the experimental results: the sum of coordination numbers around Pt is small (**Tables S10–13**) and the MSDs of Pt have small offsets (**Figures S21–24**). However, although N is correlated with the static offset of $\sigma^2(T)$, the temperature dependence of $\sigma^2(T)$ is not significantly affected by the correlation as discussed in the Supporting Information Section **3.7**. It holds true in the case of the spectral overlap issue. The Einstein temperature of the contaminated spectra was estimated to be 175.8(3) K, whose error compared with the genuine value (178 K) was approximately 1%. To conclude, the spectral overlap would result in small enough errors of the atomic radii and Einstein temperatures, which were mainly discussed in this article. However, coordination numbers and static strains would be susceptible to the overlap, and thus further experimental improvements to avoid the overlap, such as K-edge measurements or range-extended EXAFS, are required to enable reliable estimation of these quantities.

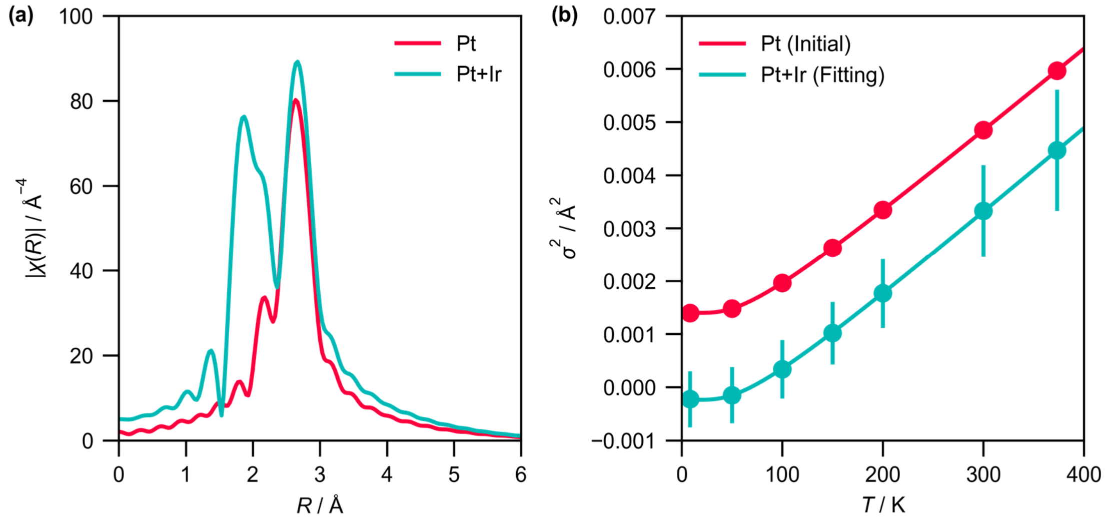

Figure S31 **(a)** The simulated FT-EXAFS of Pt monometal (Pt) and the synthesized spectra of Pt and Ir (Pt+Ir) at 8 K. **(b)** The $\sigma^2(T)$ used in the simulation of Pt L_3 EXAFS (Pt (Initial)) and those estimated by fitting the Pt+Ir spectra EXAFS (Pt+Ir (Fitting)).

Table S16 The parameters used in the EXAFS simulation of the EXAFS of Pt (Pt (Initial)), and the corresponding values estimated by fitting the simulated Pt+Ir spectra (Pt+Ir (Fitting)).

	Pt (Initial)	Pt+Ir (Fitting)
N	12	8(1)
ΔE_0 / eV	0	6(2)
R / Å	2.766	2.802(5)
$\sigma^2(8\ \mathrm{K})$ / 10^{-3} Å^2	1.4	–0.2(5)
$\sigma^2(50\ \mathrm{K})$ / 10^{-3} Å^2	1.5	–0.1(5)
$\sigma^2(100\ \mathrm{K})$ / 10^{-3} Å^2	2.0	0.3(6)
$\sigma^2(150\ \mathrm{K})$ / 10^{-3} Å^2	2.6	1.0(6)
$\sigma^2(200\ \mathrm{K})$ / 10^{-3} Å^2	3.3	1.8(6)
$\sigma^2(300\ \mathrm{K})$ / 10^{-3} Å^2	4.8	3.3(9)
$\sigma^2(373\ \mathrm{K})$ / 10^{-3} Å^2	6.0	4(1)
θ_E / K	178	175.8(3)

§4. Computational details

§4.1. Lattice Parameters

The estimated lattice parameters (**Figure S32**) were used to estimate atomic radii of elements using **Eq. 6**. The corresponding analyses were performed using two different datasets: the first included all 64 alloys, whereas the other included 40 alloys with X = PGM. **Figure S33** shows the results of linear regression analysis for each dataset. The results indicated that the behaviors of PGM- and *p*M-rich alloys cannot be described using a single linear model. In contrast, the lattice parameters of PGM-rich alloys are well described by **Eq. 6**. The latter dataset would better represent our MEA NPs, considering the PGM to *p*M ratios; therefore, the further discussion refers to the latter result. **Table S17** summarizes the atomic radii estimated using the PGM-rich alloy dataset. Although the values of PGMs were almost identical to the atomic radii in the corresponding monometals, the values of *p*Ms were notably smaller than those in the corresponding monometals. The results help explain the atomic radii estimated by XRD and EXAFS analyses.

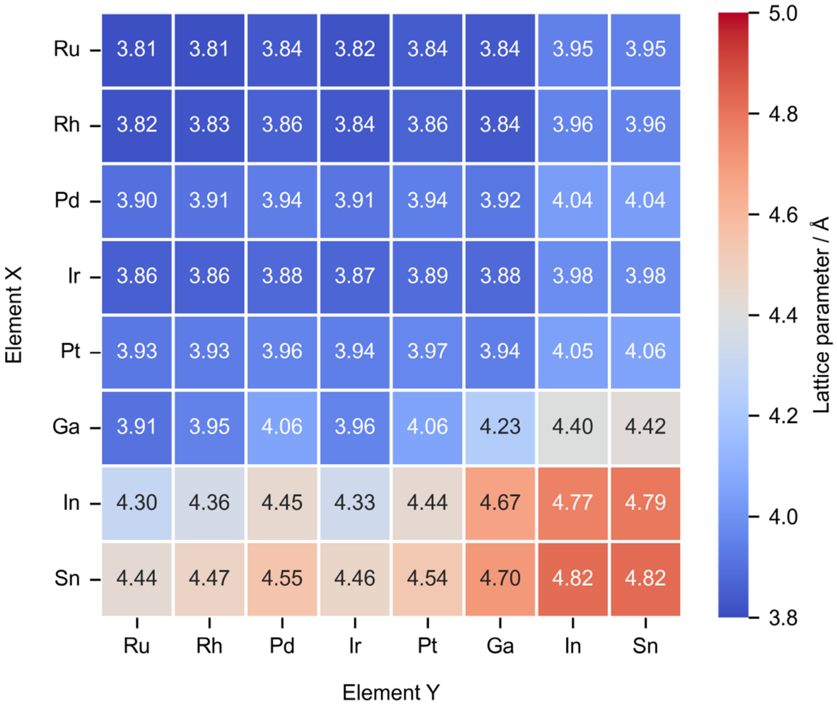

Figure S32 Lattice parameters (Å) of a series of $L1_2$ alloys X_3Y. The diagonal components (X = Y) are the values of fcc monometals.

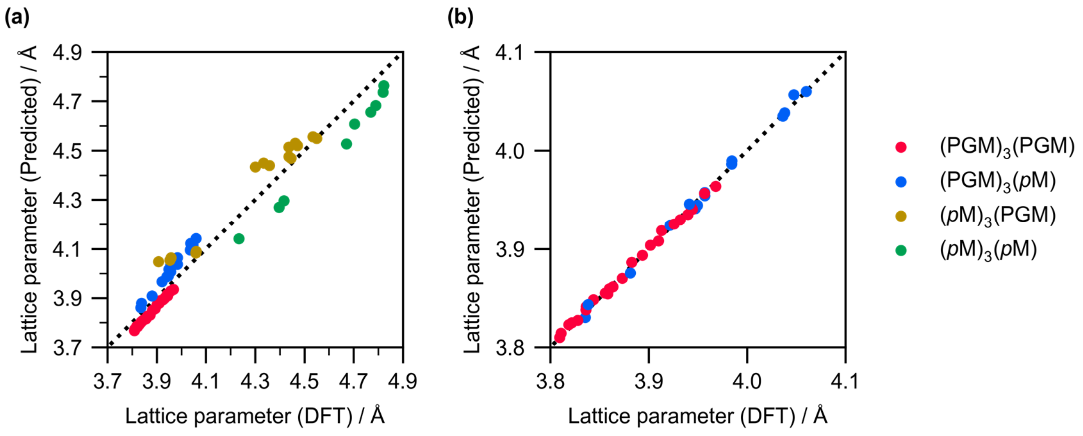

Figure S33 Lattice parameters of alloys calculated using DFT and predicted by linear regression based on **Eq. 6**. The datasets used in **(a)** encompass all the 64 alloys, while only PGM-rich alloys are selected in **(b)**. The markers in different colors represents the alloys where X and Y belong to different element groups (PGM or *p*M).

Table S17 Atomic radii (Å) of different elements estimated using the linear regression based on **Eq. 6** (column: Alloy) and those in the monometals calculated by dividing the monometal lattice parameters by $2\sqrt{2}$ (column: Monometal).

	**Alloy / Å**	**Monometal / Å**
**Ru**	1.3469(7)	1.3469
**Rh**	1.3531(7)	1.3534
**Pd**	1.3912(7)	1.3929
**Ir**	1.3682(7)	1.3694
**Pt**	1.4014(7)	1.4029
**Ga**	1.376(3)	1.4971
**In**	1.533(3)	1.6865
**Sn**	1.537(3)	1.7053

§4.2. Einstein Temperatures

To discuss Einstein temperatures, we calculated the total energies of the structures with one-atom displacements. Herein, the 2 × 2 × 2 supercells were used as models to suppress interactions between the displaced atoms. Displacements were applied to one atom in the supercell along the bond with its nearest neighbor (along the <110> direction). The displacement scales were 2%, 4%, and 6 % of the first-nearest-neighbor distances. Calculations were conducted for 1) a Y atom displaced along a Y–X bond, and 2) an X atom displaced along an X–Y bond. Note that these patterns are identical in fcc monometals. The potential force constant k was derived assuming a harmonic potential (**Figure S34**), and the Einstein temperatures were calculated using **Eq. 2** and are summarized in **Figure S35**. *p*M-rich alloys (X = *p*M and Y = PGM) showed anharmonic (Ga_3Pd), double-well-shaped (Ga_3Ir and Ga_3Pt), or convex upward (the others) potentials when a Y atom was displaced, which indicates structural instability; therefore, the values are not shown in **Figure S35a**.

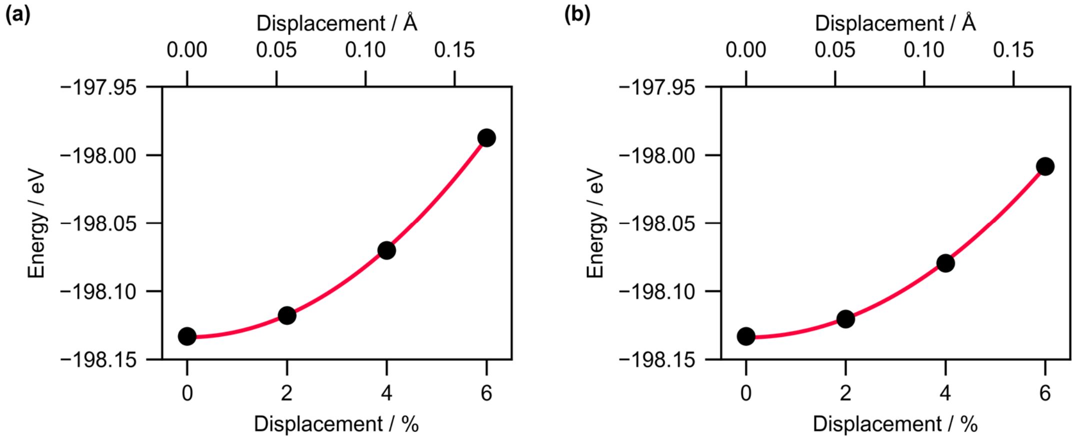

Figure S34 Energy curves of a Rh_3In 2 × 2 × 2 supercell with one-atom displacements as an example. The displacements were applied **(a)** to an In atom along an In–Rh bond and **(b)** to a Rh atom along a Rh–In bond. Red lines represent fitting curves obtained assuming a harmonic potential.

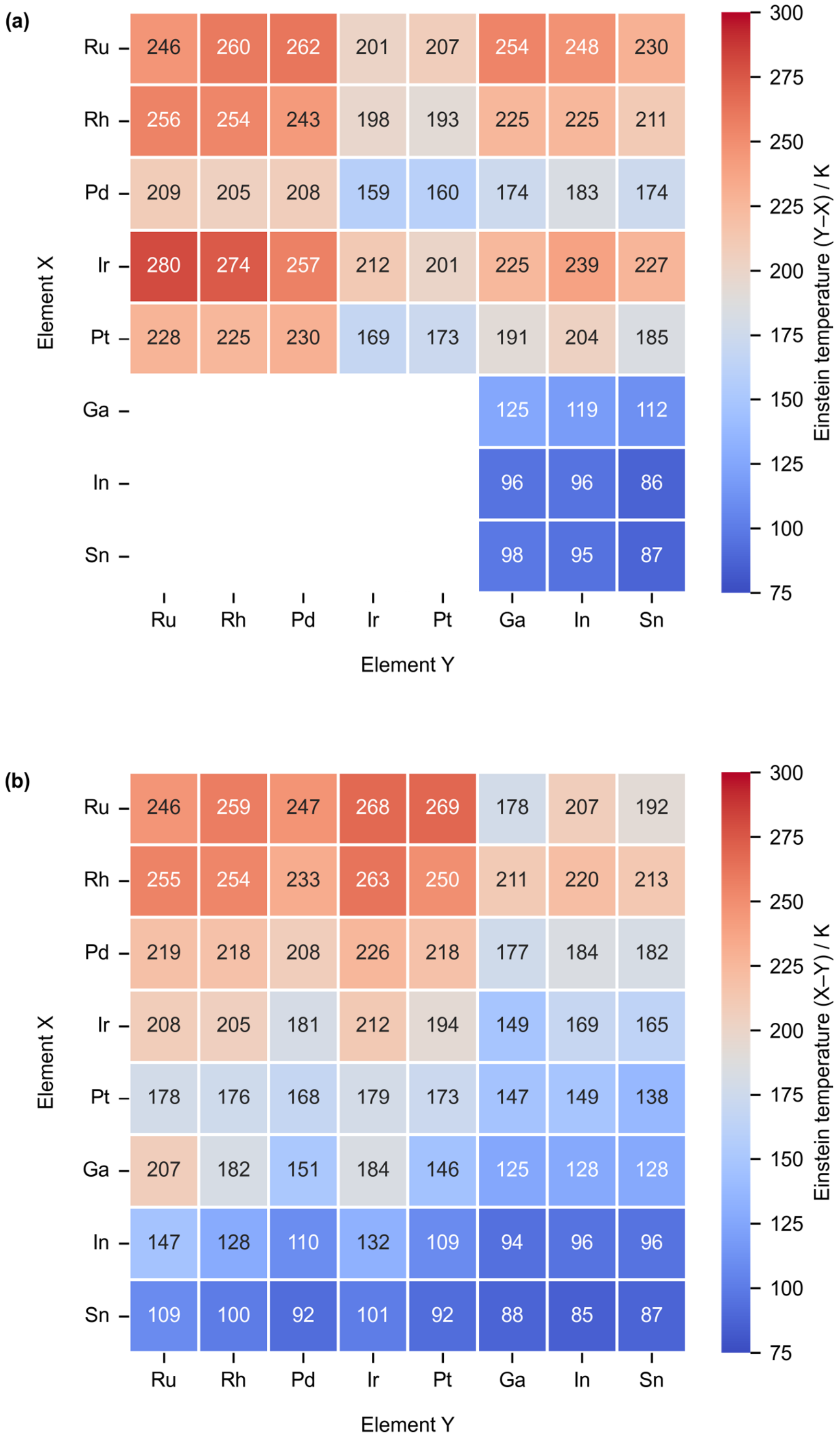

Figure S35 Einstein temperatures (K) of a series of $L1_2$ alloys X_3Y estimated by displacing **(a)** a Y atom along a Y–X bond and **(b)** an X atom along a X–Y bond. The diagonal components (X = Y) are the values of fcc monometals.

§4.3. DOS Calculations

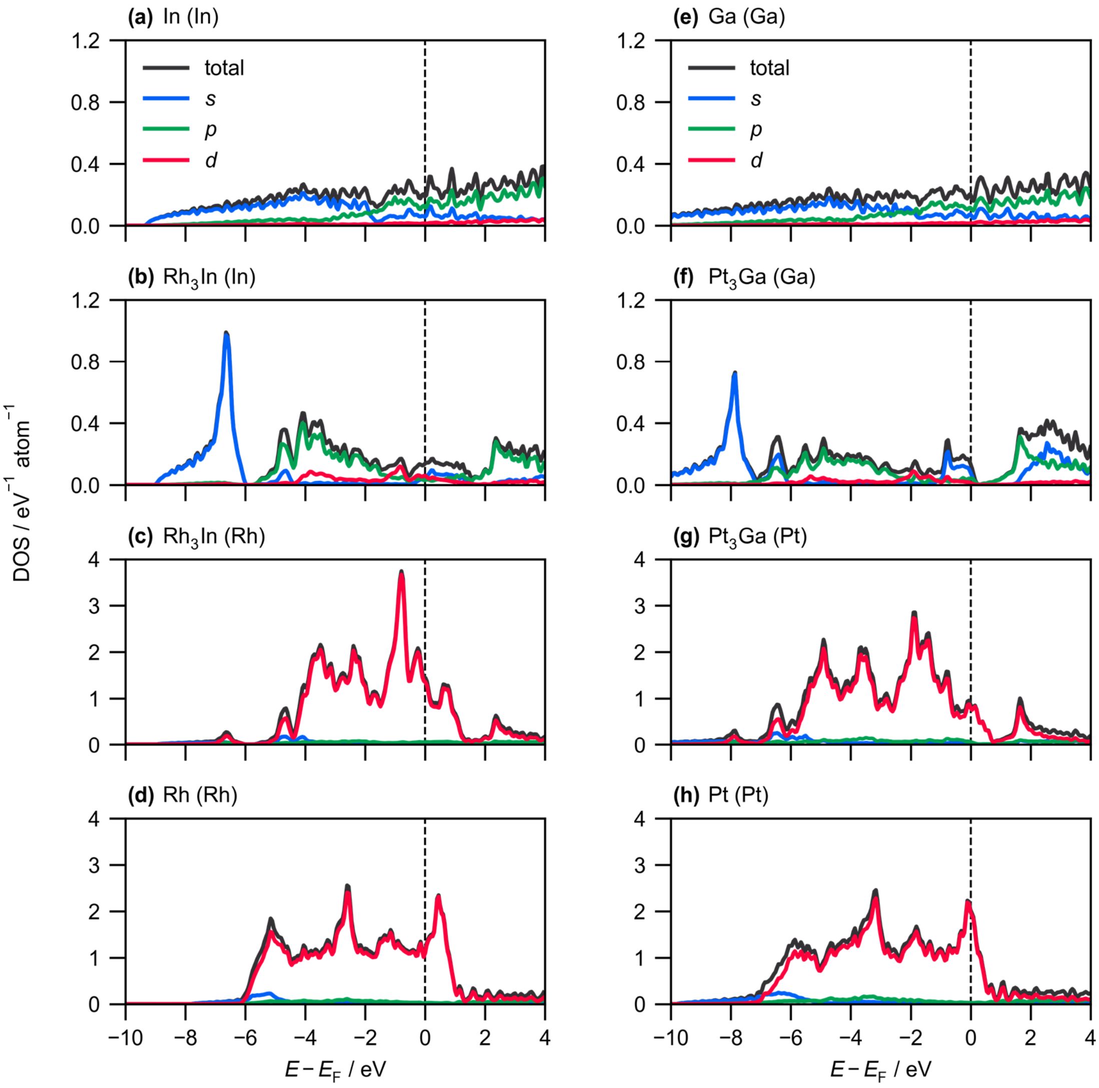

Figure S36 **(a–d)** Local DOSs of Rh_3In projected onto (b) In and (c) Rh orbitals, and local DOSs of the corresponding fcc monometals ((a) In and (d) Rh). **(e–h)** Local DOSs of Pt_3Ga projected onto (f) Ga and (g) Pt orbitals, and local DOSs of the corresponding fcc monometals ((e) Ga and (h) Pt).

§4.4. Formation Energy

The formation energies of the L1$_2$ alloys were defined as

$$\Delta E_\mathrm{f}(\mathrm{X_3Y}) = E(\mathrm{X_3Y}) - \frac{3}{4}E(\mathrm{X}) - \frac{1}{4}E(\mathrm{Y})$$

where $E(\mathrm{alloy})$ is the total energy of the alloy.

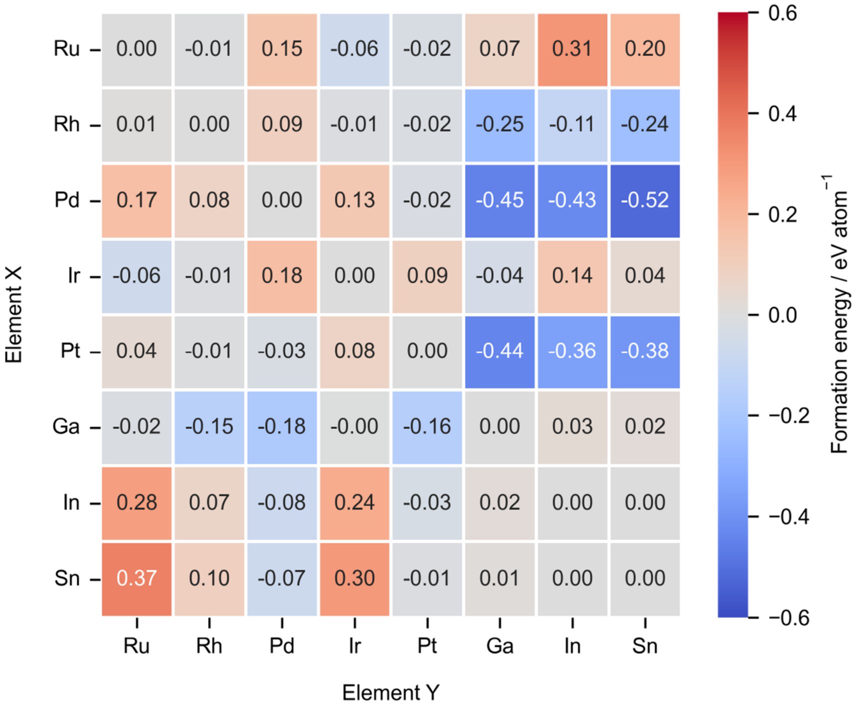

Figure S37 Estimated formation energies (eV/atom) of a series of L1$_2$ alloys X_3Y. The diagonal components (X = Y) are the values of fcc monometals.

§5. Literature Values of Monometals

Table S18 Previously reported atomic radii,[13–18] melting points,[13] Brinell hardnesses,[19] and electronegativities[20] (Pauling scale) of monometals. The atomic radius was calculated as a half of the first-nearest-neighbor distance. For Ga and Sn, the atomic radii were calculated based on the structures of different polymorphs.

	Atomic radius / Å	Melting point / °C	Brinell hardness / MPa	Electronegativity
**Ru**	1.33	2334	2160	2.2
**Rh**	1.34	1964	981-1350	2.2
**Pd**	1.38	1555	320-610	2.2
**Ir**	1.36	2446	1670	2.2
**Pt**	1.39	1768	310-500	2.2
**Ga**	α: 1.22<br>β: 1.34<br>γ: 1.30<br>δ: 1.28<br>II: 1.39<br>III: 1.41	30	56.8-68.7	1.6
**In**	1.63	157	8.83-10	1.7
**Sn**	α: 1.41<br>β: 1.51	232	51-75	1.8

▼References

(1) Schneider, C. A.; Rasband, W. S.; Eliceiri, K. W. NIH Image to ImageJ: 25 Years of Image Analysis. *Nat. Methods* **2012**, *9* (7), 671–675.

(2) Gorham, J. NIST X-Ray Photoelectron Spectroscopy Database - SRD 20, 2012. https://doi.org/10.18434/T4T88K.

(3) Ravel, B.; Newville, M. ATHENA, ARTEMIS, HEPHAESTUS: Data Analysis for X-Ray Absorption Spectroscopy Using IFEFFIT. *J. Synchrotron Radiat.* **2005**, *12* (4), 537–541.

(4) Toby, B. H.; Von Dreele, R. B. *GSAS-II*: The Genesis of a Modern Open-Source All Purpose Crystallography Software Package. *J. Appl. Crystallogr.* **2013**, *46* (2), 544–549.

(5) Fornasini, P.; Grisenti, R. On EXAFS Debye-Waller Factor and Recent Advances. *J. Synchrotron Radiat.* **2015**, *22* (5), 1242–1257.

(6) Maruta, Y.; Kusada, K.; Wu, D.; Yamamoto, T.; Toriyama, T.; Matsumura, S.; Seo, O.; Yasuno, S.; Kawaguchi, S.; Sakata, O.; Kubota, Y.; Kitagawa, H. Compositional Dependence of Structures and Hydrogen Evolution Reaction Activity of Platinum-Group-Metal Quinary RuRhPdIrPt Alloy Nanoparticles. *Chem. Commun.* **2022**, *58* (44), 6421–6424.

(7) Stern, E. A. Number of Relevant Independent Points in X-Ray-Absorption Fine-Structure Spectra. *Phys. Rev. B* **1993**, *48* (13), 9825–9827

(8) Newville, M. Larch: An Analysis Package for XAFS and Related Spectroscopies. *J. Phys.: Conf. Ser.* **2013**, *430*, 012007.

(9) McMaster, W. H.; Del Grande, N. K.; Mallett, J. H.; Hubbell, J. H. Compilation of X-Ray Cross Sections; UCRL-50174 (Section 2) (Revision 1); California University, Livermore. Lawrence Radiation Laboratory, 1969.

(10) Zabinsky, S. I.; Rehr, J. J.; Ankudinov, A.; Albers, R. C.; Eller, M. J. Multiple-Scattering Calculations of X-Ray-Absorption Spectra. *Phys. Rev. B* **1995**, *52* (4), 2995–3009.

(11) Newville, M.; Līviņš, P.; Yacoby, Y.; Rehr, J. J.; Stern, E. A. Near-Edge X-Ray-Absorption Fine Structure of Pb: A Comparison of Theory and Experiment. *Phys. Rev. B* **1993**, *47* (21), 14126–14131.

(12) Beni, G.; Platzman, P. M. Temperature and Polarization Dependence of Extended X-Ray Absorption Fine-Structure Spectra. *Phys. Rev. B* **1976**, *14* (4), 1514–1518.

(13) Lide, D. R. *CRC Handbook of Chemistry and Physics, 85th Edition*; CRC Press: Boca Raton, Florida, 2004.

(14) Wyckoff, R. W. G. *Crystal Structures, Volume 1*, *2nd Edition*; Interscience Publishers, 1963.

(15) Bosio, L.; Defrain, A.; Curien, H.; Rimsky, A. Structure Cristalline du Gallium β. *Acta Crystallogr. Sect. B* **1969**, *25* (5), 995–995.

(16) Bosio, L.; Curien, H.; Dupont, M.; Rimsky, A. Structure Cristalline de Ga γ. *Acta Crystallogr. Sect. B* **1972**, *28* (6), 1974–1975.

(17) Bosio, L.; Curien, H.; Dupont, M.; Rimsky, A. Structure Cristalline de Gaδ. *Acta Crystallogr. Sect. B* **1973**, *29* (2), 367–368.

(18) Bosio, L. Crystal Structures of Ga(II) and Ga(III). *J. Chem. Phys.* **1978**, *68* (3), 1221–1223.

(19) Samsonov, G. V. Mechanical Properties of the Elements. In *Handbook of the Physicochemical Properties of the Elements*; Samsonov, G. V., Ed.; Springer US: Boston, MA, 1968.

(20) Pauling, L. *The Nature of the Chemical Bond, 3rd Edition*; Cornell University Press: Ithaca, NY, 1960.
